# Heavy metal contamination in the complete stretch of Yamuna river: A fuzzy logic approach for comprehensive health risk assessment

**DOI:** 10.1371/journal.pone.0272562

**Published:** 2022-08-08

**Authors:** Maneesh Jaiswal, Sanjay Kumar Gupta, Mayuri Chabukdhara, Mahmoud Nasr, Arvind Kumar Nema, Jakir Hussain, Tabarak Malik

**Affiliations:** 1 Department of Civil Engineering, Indian Institute of Technology Delhi, New Delhi, India; 2 Department of Environmental Biology and Wildlife Sciences, Cotton University, Guwahati, Assam, India; 3 Environmental Engineering Department, Egypt-Japan University of Science and Technology (E-JUST), Alexandria, Egypt; 4 Faculty of Engineering, Sanitary Engineering Department, Alexandria University, Alexandria, Egypt; 5 Upper River Yamuna Board, Department of Water Resources, River Development and Ganga Rejuvenation, New Delhi, India; 6 Department of Biomedical Sciences, Institute of Health, Jimma University, Jimma, Ethiopia; Yangtze Normal University, CHINA

## Abstract

River Yamuna is one of the most sacred major tributaries of river Ganga. This study aimed to assess the level of heavy metals in monsoon and non-monsoon season in river Yamuna in Uttar Pradesh, India and to assess the possible source of contamination and its associated health risk. Except for iron (Fe), the mean levels of all metals were within drinking water safe limits in both seasons. Except for chromium (Cr), lower values were observed for other metals in the monsoon season could be attributed dilution effect. Multivariate analysis indicated that both geogenic and anthropogenic sources contribute to heavy metals in river Yamuna in monsoon and non-monsoon seasons. The health risk in terms of hazard index (HI) and fuzzy-logic hazard index (FHI) demonstrated that both HI and FHI values among children exceeded the safe limit in most of the sites in non-monsoon seasons and in few in monsoon season. For adults, HI and FHI values were within safe limit.

## 1. Introduction

River water have been used for various purposes including drinking, irrigation, domestic and industrial applications [1, 2]. Unfortunately, the water quality in rivers has recently suffered from dramatic deterioration due to various anthropogenic and natural activities [3]. Among different pollutants, heavy metals are of serious concern due to their toxic, bioaccumulative, non-biodegradable nature [4].

Arsenic (As), cadmium (Cd), chromium (Cr) and lead (Pb) rank among the priority metals that are of great public health significance and are also classified as either “known” or “probable” human carcinogens based on epidemiological and experimental studies [5]. Other complications associated with heavy metals include gastrointestinal and kidney dysfunction, nervous system disorders, skin lesions, vascular damage, immune system dysfunction and birth defects [6]. The International Agency for Research on Cancer (IARC) has classified nickel as a potentially carcinogenic substance [7]. Metals such as zinc, copper and iron are essential elements that are required for several chemical or biochemical processes in the body but are toxic above a certain concentration [8]. In the past few decades the concentration of metals in almost all the Indian rivers has increased due to anthropogenic activities [9–11]. Heavy metals from industrial effluents and surface and agricultural runoff have been considered a major source of water pollution, causing serious human health risks [12, 13]. Accordingly, comprehensive investigations should be performed to assess the health risks associated with human exposure to metal-contaminated water, providing sustainable strategies for managing the river systems.

India’s population relies on the River Yamuna as the primary water source for domestic purposes and agricultural applications, fulfilling more than 90% of the total water demand in several districts [14]. Unfortunately, in recent years the Indian Yamuna river and its major tributaries and catchment area have suffered from severe pollution due to the discharge of untreated or partially treated wastewater containing undesirable levels of toxic metals [2]. Hence, the study objectives are four fold: (1) to analyze the toxic elements along the entire Yamuna stretch for the monsoon and non-monsoon seasons compared with the national and international standards, (2) to assess their possible sources by using multivariate analysis, (3) estimate potential noncarcinogenic human health risk for adults and children, (4) employ a fuzzy-logic approach to generate a new criterion for health risk forecasting, namely fuzzy-logic hazard index (FHI).

## 2. Materials and methods

### 2.1. Study area and water sampling

The Yamuna is one of the largest and longest rivers in India, with a total length of about 1376 km and a catchment area of 366,223 km^2^. It originates from the Yamunotri glacier (38° 59′ N, 78° 27′ E) of the lower Himalayas in the District Uttarkashi (Uttranchal). The river can be classified into five sectors: the Himalayan, Upper, Delhi, Eutrophicated, and Diluted segments, as demonstrated by Sharma and Kansal [14]. The important geo-environmental conditions of the River Yamuna and the associated catchment basins are listed in the S1 Table. No special permission was needed for collecting the samples from all along the stretch of river Yamuna.

**Fig 1:**
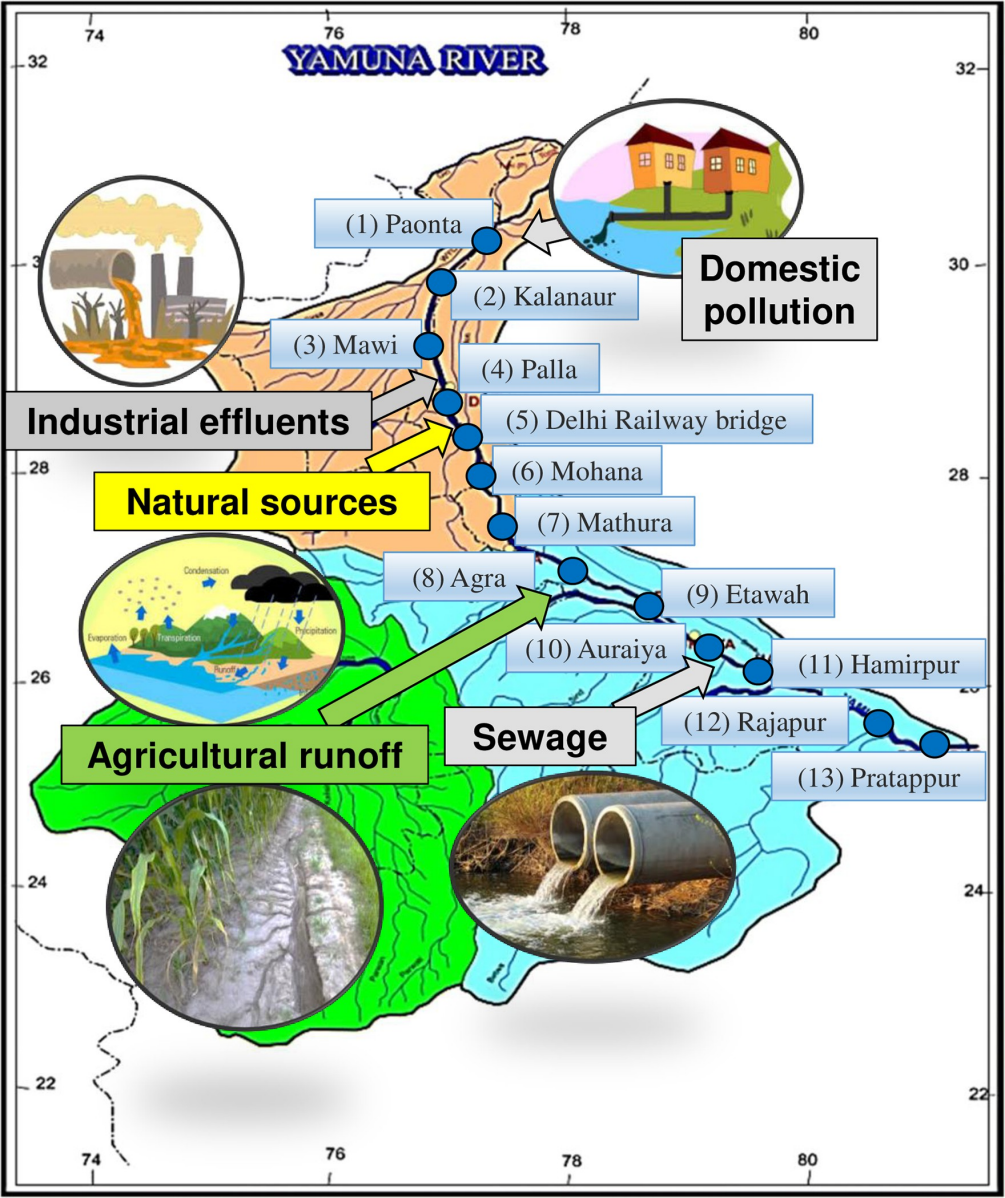
Map showing river Yamuna, its tributaries, and sampling locations.

In this study, 13 sampling sites were selected to monitor and assess the distribution of metal pollution along the entire Yamuna stretch. These sites corresponded to 13 districts, namely Poanta, Kalanaur, Mawi, Palla, Delhi, Mohana, Mathura, Agra, Etawah, Auraiya, Hamirpur, Rajapur, and Pratappur (Fig 1). The sampling sites and location details are given in S2 Table. The water sampling procedure was performed twice a year, during the monsoon (July to October) and non-monsoon (November to June) seasons from 2011 to 2018. One liter of water samples was collected from 30 to 50 cm depth using the grab method from the middle of the river. During non-monsoon, there is no or limited rainfall, and river water levels decrease; during monsoon, river water levels increase due to heavy rain. The variation in rainfall during monsoon and non-monsoon seasons can cause the variation of metals concentration in water.

After collection, the samples were filtered, acidified, and preserved at 4°C in an icebox, following Gupta et al. [10]. Eight metals, i.e., arsenic (As), cadmium (Cd), chromium (Cr), copper (Cu), nickel (Ni), lead (Pb), iron (Fe), and zinc (Zn), were selected in this study to assess the human health risk along the river.

### 2.2 Analytical analysis

The water samples were acid digested for metal analysis by adding 20 mL of concentrated HNO_3_ at 100°C until dryness [15]. The respective digests were cooled to room temperature, diluted, and filtered by Whatman no. 42 filter paper, following Gupta et al. [10]. Further, the atomic absorption spectrometry (Varian AA240 Zeeman, USA) was used to measure the metal concentrations of each digest. All reagents and chemicals used in this study were of analytical grade and procured from E. Merck Ltd., India. Ultrapure water (18.2 MΩ cm at 25°C; USF Elga, Germany) was used throughout the study to prepare all standards. Certified stock solutions purchased from E. Merck Ltd., Germany, were used for preparing the calibration curves. Quality control of the analytical analysis was guaranteed by the use of standard operating procedures (SOP), reagent blanks, reagent set spike samples, and recovery of spiked control replicate.

### 2.3 Data analysis

The relationship between various metals was analyzed using Pearson’s correlation coefficient. Correlation analysis was performed to understand the relationship among metal pairs. Principal component analysis (PCA) was carried out to identify the principal sources of variation in the data set due to interrelated variables [16]. Two multivariate statistical techniques were employed, the PCA and the HCA (Hierarchical Cluster Analysis). The details of PCA and HCA are mentioned elsewhere [17]. The similarity and variation among various sites were determined by Cluster analysis. Before performing the FA/PCA, the dataset was first standardized to avoid numerical ranges of the original variables. The data were analyzed using a statistical package SPSS^®^ (Window Version 17.0), and with XlStat, an add-in package of Microsoft Excel 2011.

#### 2.3.1 Health risk assessment

In this study, the magnitude, frequency, and duration of human exposure to metals in the River Yamuna were used to estimate the non-cancer health risk. For this purpose, the average daily dose (ADD) was derived from Eq 1 [18].

$$\mathrm{ADD}=\frac{C\times \mathrm{IR}\times \mathrm{EF}\times \mathrm{ED}}{\mathrm{BW}\times \mathrm{AT}}$$ (Eq 1)

where, ADD is the average daily dose (mg/kg/day), C is the mean concentration of metal (mg/L), IR is the intake rate of metal-contaminated water (3.45 L/day for adults and 2.0 L/day for children), EF is the exposure frequency (365 days/year), ED is the exposure duration (70 years for adults and 10 years for children), BW is the average body weight (60 kg for adults and 25 kg for children), and AT is the average time (25,550 days for adults and 3,650 days for children).

HQ expresses the potential exposure to an element divided by the appropriate chronic or acute dose that has no adverse effects [19]. HQ of an individual metal in the dose-response assessment was calculated by Eq (2).

$$\mathrm{HQ}=\frac{\mathrm{ADD}}{\mathrm{RfD}}$$ (Eq 2)

where, RfD is the oral reference dose in mg/kg/day considered as 3.0E−04 (As), 1.0E−03 (Cd), 3.0E−03 (Cr), 3.7E−02 (Cu), 2.0E−02 (Ni), 3.5E−03 (Pb), 7.0E−01 (Fe), and 3.0E−01 (Zn).

HI defines the total hazard of all constituents in a mixture of toxics, affecting a specific route/pathway [19]. HI was computed from Eq (3) by the summation of individual HQs of each metal.


HI=∑HQ (Eq 3)

For the risk assessment of a mixture of elements, if the value of HQ and/or HI exceeds 1, there could be potential noncarcinogenic effects on human health. The HQ and HI criteria were calculated for two population groups, i.e., adults and children.

#### 2.3.2. Fuzzy logic-based model for health risk assessment

*2*.*3*.*2*.*1 Fuzzy model concept*. The fuzzy logic approach was initiated by Zadeh [20] to represent intrinsically vague or linguistic knowledge using a set of If-Then inference rules, i.e., “IF X AND Y THEN Z”. Generally, the number and quality of the rules affect the robustness of the system under study. Models based on fuzzy rules could extract relevant information from uncertain and inaccurate data, depending on the human inference process and knowledge [21]. Hence, the fuzzy-based classification allows for logical, reliable, and transparent information of data collection by expressing multiple levels within the scale (0–1), i.e., instead of only two levels (0 or 1) in classical clustering.

**Fig 2:**
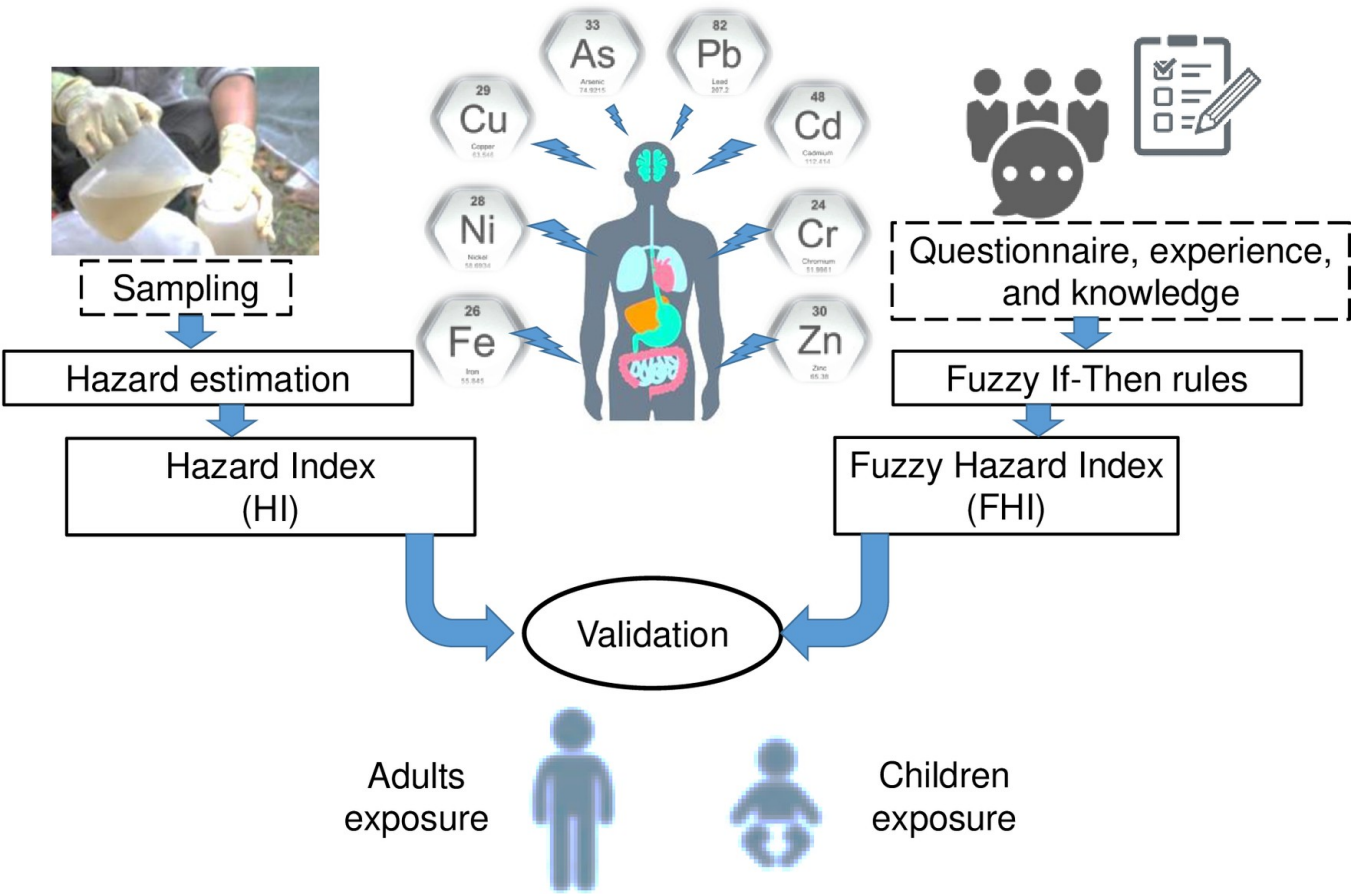
Framework for HI and FHI estimations.

In this study, fuzzy logic was employed to describe the human health risk associated with exposure to the trace metals in the River Yamuna (Fig 2). The parameters of the fuzzy-based model used to predict the fuzzy-logic hazard index (FHI) were selected based on personal knowledge, experience, and understanding of the metal-health risk relationship. For this purpose, a Mamdani-type fuzzy model was employed to represent concise correlations between eight metals (As, Cd, Cr, Cu, Ni, Pb, Fe, and Zn) and FHI. Each input was classified into three categories (cluster1, cluster2, and cluster3), using the Gaussian curve membership function. The parameters of the Gaussian shape functions, along with the linguistic classification of FHI for adult and children, are given in S3 and S4 Tables. The amount of overlap of membership functions for each input variable was assigned by an expert’s advice and the permissible limit of each metal [3]. Three fuzzy If-Then rules were considered to be suitable for this study. The ranges of the fuzzy sets for each variable were selected, following the methodologies performed elsewhere [12, 22, 23].

**Fig 3:**
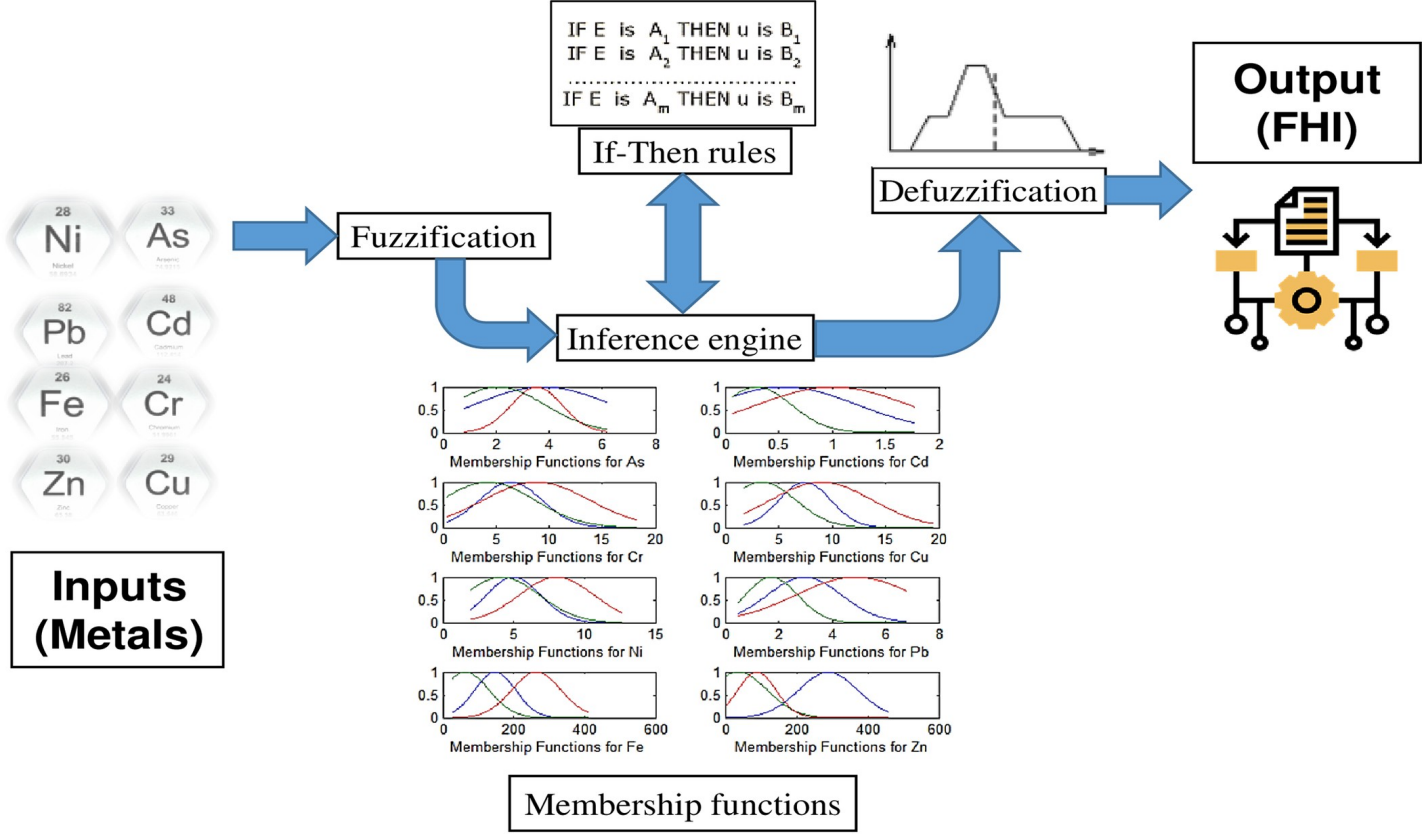
Fuzzy model procedures for FHI estimation: (i) Fuzzification (Fuzzify inputs); (ii) Inference Engines or Fuzzy If-Then Rules; (iii) Defuzzification.

*2*.*3*.*2*.*2 Fuzzy-model procedures*. The fuzzy logic-based index was developed by conducting three major steps, which can be described as follows (Fig 3):

***(i) Fuzzification (Fuzzify inputs)*:**

In the Fuzzification process, each non-fuzzy value is transferred into a number between 0 and 1 through a membership function. The Gaussian shape was selected in this study as the appropriate membership function for all variables (input and output). For instance, the first input, “As” was classified into three fuzzy linguistic sets: in1cluster1, in1cluster2, and in1cluster3 (S3 and S4 Tables).

***(ii) Inference Engines or Fuzzy If-Then Rules*:**

In the second step, three If-Then inference rules were created to map conceivable relationships between the input and output variables. The first rule can be enunciated simply as follows:

If the inputs to the FIS, i.e., As, Cd, Cr, Cu, Ni, Pb, Fe, and Zn, strongly belong to their respective cluster membership functions, then the output (FHI) must strongly belong to its cluster membership function. In the fuzzy description, this rule can be transformed into an If-Then fuzzy rule:

*Rule 1*: IF (As is in1cluster1) AND (Cd is in2cluster1) AND (Cr is in3cluster1) AND (Cu is in4cluster1) AND (Ni is in5cluster1) AND (Pb is in6cluster1) AND (Fe is in7cluster1) AND (Zn is in8cluster1) THEN (FLHI is out1cluster1) (1).

In the same way, the other two rules used to define the behavior of the system are:

*Rule 2*: IF (As is in1cluster2) AND (Cd is in2cluster2) AND (Cr is in3cluster2) AND (Cu is in4cluster2) AND (Ni is in5cluster2) AND (Pb is in6cluster2) AND (Fe is in7cluster2) AND (Zn is in8cluster2) THEN (FLHI is out1cluster2) (1).

*Rule 3*: IF (As is in1cluster3) AND (Cd is in2cluster3) AND (Cr is in3cluster3) AND (Cu is in4cluster3) AND (Ni is in5cluster3) AND (Pb is in6cluster3) AND (Fe is in7cluster3) AND (Zn is in8cluster3) THEN (FLHI is out1cluster3) (1).

Each rule has a weight, which can take a value between 0 and 1. In this study, the (1) at the end of the rule indicates that the rule has a weight or importance of "1".

The fuzzy operator was employed to give one number that denotes the result of the rule antecedent (i.e., the “If” part of each rule), covering the eight input attributes. The logical “AND” operator (the minimum of the options) was applied to the antecedent, followed by the implication method “MIN” to truncate the output membership function. This output is known as the consequent of the rule (i.e., the “Then” part of each rule).

***(iii) Defuzzification*:**

The consequents of all rules are combined into a single fuzzy set via the “MAX” (maximum) aggregation method. Other aggregation tools, including “PROBOR” (probabilistic OR) or “SUM” (sum of the rule output sets), could also be selected based on the fuzzy model application. Finally, the result of aggregation was subjected to a defuzzification process to obtain the final decision (a single number). The defuzzification method used was the Centroid calculation, whereas other mathematical techniques, including Bisector, Largest of maximum, Middle of maximum, and Smallest of maximum, could also be adopted. The centroid method was selected for defuzzification since it is the most prevalent and physically appealing to various model structures.

All the computations were processed using the “fuzzy logic toolbox” in MATLAB R2013a (http://www.mathworks.com/).

## 3. Results and discussion

### 3.1 Trace and toxic elements in water

In this study, water samples were collected from 13 districts distributed along the River Yamuna stretch and analyzed for metals (As, Cd, Cr, Cu, Ni, Pb, Fe, and Zn). Statistical summary of metal concentrations in river Yamuna in monsoon and non-monsoon seasons are shown in Table 1.

**Table 1:** 

	As	Cd	Cr	Cu	Ni	Pb	Fe	Zn
**Monsoon season**								
Minimum	0.790	0.072	0.385	1.803	1.965	0.485	26.933	4.933
Maximum	6.213	0.875	18.280	19.470	10.975	3.953	412.000	279.267
Mean	2.553	0.300	7.451	5.374	4.790	1.800	117.354	45.272
SD	1.700	0.238	6.276	5.352	2.820	0.903	112.853	74.282
**Non-monsoon season**								
Minimum	0.893	0.110	2.429	2.518	3.078	0.885	47.643	13.350
Maximum	4.660	1.776	10.300	8.713	12.701	6.808	303.286	459.953
Mean	3.232	0.621	5.118	6.141	5.405	3.213	145.345	192.023
SD	1.174	0.571	2.397	1.945	2.512	2.035	92.395	130.077

**Fig 4:**
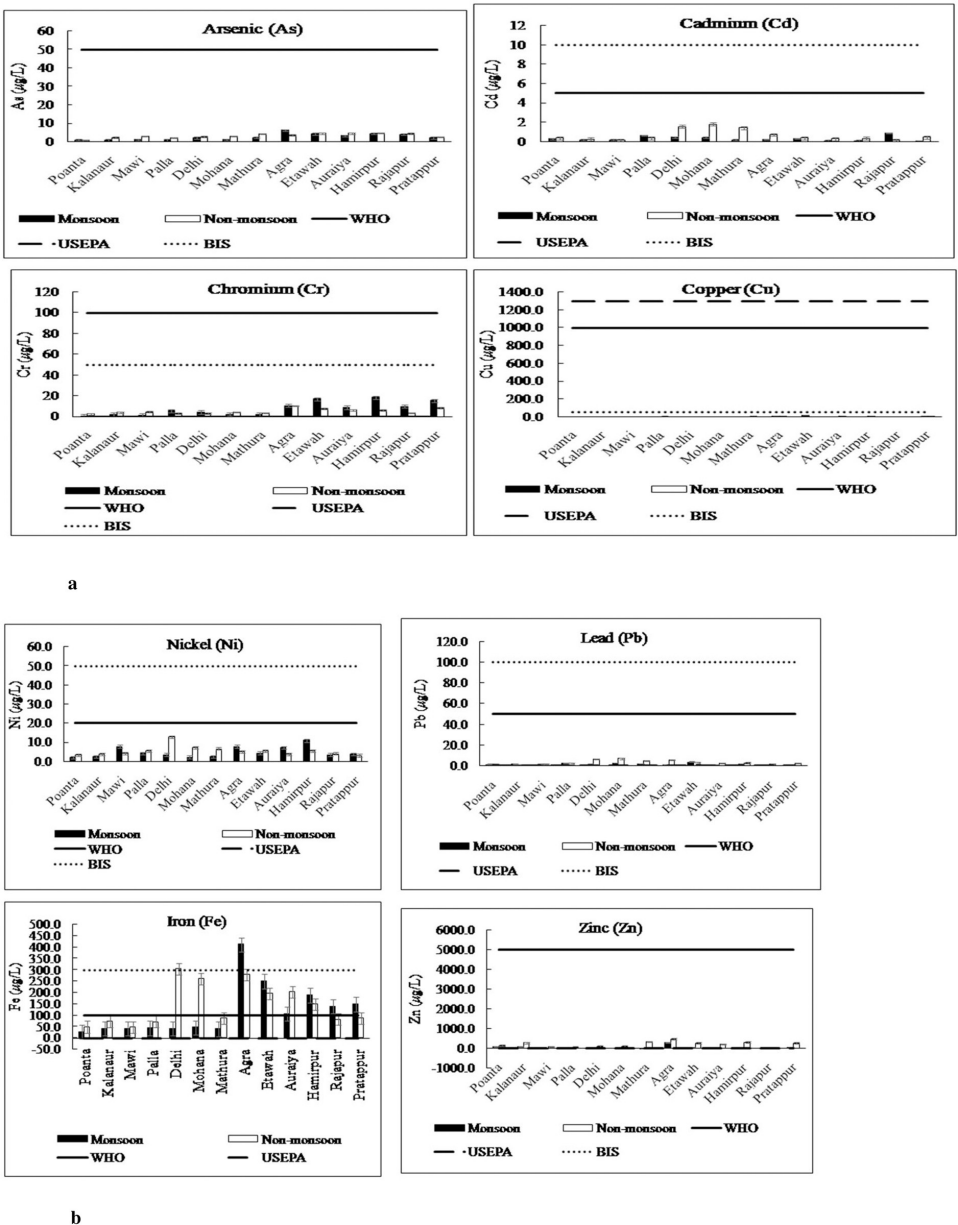
**a.** Mean concentrations of As, Cd, Cr and Cu in different sites of river Yamuna. **b.** Mean concentrations of Ni, Pb, Fe and Zn in different sites of river Yamuna.

The mean concentrations of 8 metals in monsoon and non-monsoon seasons in all the districts and its comparison with permissible limits are shown in Fig 4A and 4B. The level of pollution of each element and the associated health concern is given as follows:

#### 3.1.1. Arsenic

In this study, the As ranges were 0.79–6.21 μg/L for monsoon and 0.89–4.66 μg/L for non-monsoon, with mean values of 2.55 and 3.23 μg/L, respectively (Table 1). The results depicted that the mean As concentrations were within the acceptable limits in both the seasons (Fig 4A). The highest As level was reported in Agra during the monsoon season and Hamirpur during the non-monsoon season. Arsenic comes into water from weathering and leaching of rocks, arsenical pesticides, fertilizers, and disposal of industrial and animal wastes [24]. Arsenic contamination in river Yamuna in Delhi has also been linked to coal-based thermal power plants [25]. Similarly, in another study in river Yamuna, the maximum As level reported was 6 μg/L [26].

#### 3.1.2. Cadmium

The mean Cd concentrations was found to be 0.30 μg/L in monsoon and 0.62 μg/L in non-monsoon and the values were within permissible limits (Fig 4A). In another study in river Yamuna in Delhi stretch, the mean Cd level reported in monsson, pre-monsoon and post-monsoon season was 26.5 μg/L, 110.1 μg/L and 6.3 μg/L, respectively, indicating higher concentration in all seasons [27]. Similarly, Kaushik et al. [28] also reported higher Cd concentration in the range of 10 to 28 μg/L in river Yamuna in Haryana, India. In contrast, in Delhi segment of Yamuna basin, Cd was found to be below detectable limit at all locations [26]. In the present study, the water samples at the Delhi, Mohana, and Mathura regions contained higher Cd levels during the non-monsoon season. Higher levels in these districts could be attributed to use of Cd-containing fertilizers, combustion emissions, and industrial activities (e.g., mining and metal industry).

#### 3.1.3. Chromium (Cr)

The highest Cr levels during the monsoon and non-monsoon seasons were 18.28 and 10.30 μg/L, respectively. Mean Cr levels in all districts were found to be within permissible limits (Fig 4A). High Cr levels in some districts such as Pratappur, Agra, Hamirpur, and Etawah could be linked to dissolution from rain and industrial activities (e.g., electroplating, textile, metal finishing, and leather tanning). In another study in river Yamuna along Delhi stretch, Cr concentration of 60.6, 362.7 and 18.1 μg/L was reported in monsson, pre-monsson and post-monsoon season, respectively [27]. As high as 52 μg/L and 1374 μg/L of Cr was reported in Yamuna river in Delhi by Asim and Rao [29] and Sehgal et al. [26], respectively.

#### 3.1.4. Copper (Cu)

In this study, the Cu ranges were 1.80–19.47 μg/L for monsoon and 2.52–8.71 μg/L for non-monsoon, respectively. The mean values were 5.37 and 6.14 μg/L in monsoon and non-monsoon season, respectively and these values were found to be within the permissible limits (Fig 4A). However, increased Cu values in the water at Agra and Etawah may be linked to the excessive application of fungicides, fertilizers, and pesticides in irrigation, in addition to industrial activities (e.g., leather and paint production). A very higher range was reported by Sehgal et al. [26] (11–595 μg/L), Asim and Rao [29] (50–120 μg/L) and Bhardwaj et al. [27] (18.4–17642.4 μg/L) in river Yamuna at different sites.

#### 3.1.5. Nickel (Ni)

In this study, the Ni levels in River Yamuna ranged between 1.97 and 10.98 μg/L for monsoon and 3.08 and 12.70 μg/L for non-monsoon, respectively. As shown in Fig 4B, the mean Ni values (4.79 μg/L in monsoon and 5.41 μg/L in non-monsoon) complied with both Indian Standards [30] and WHO safe limits [31]. The high Ni concentration in water samples collected from Delhi could be linked to the existence of several industrial processes such as electroplating, porcelain enameling, and metal finishing. Higher mean level of Ni was reported by Bhardwaj et al. [27] in river Yamuna in monsoon (232.4 μg/L), pre-monsoon (851.5 μg/L) and post-monsoon (42.8 μg/L). Asim and Rao [29] (2021) also reported a mean level as high as 164 μg/L in river Yamuna. Higher levels of Ni was also reported in the Godavari river basin [32], *viz*., Bhatpalli (40.25 μg/L), Kumhari (24.26 μg/L), and Hivra (45.26 μg/L).

#### 3.1.6. Lead (Pb)

In this study, Pb ranged between 0.49 and 3.95 μg/L for monsoon and 0.88 and 6.81 μg/L for non-monsoon, with mean values of 1.80 and 3.21 μg/L, respectively. The mean Pb levels along the Yamuna stretch were within BIS and WHO safe limits during both seasons (Fig 4B). High Pb concentrations in the Delhi, Mohana, and Agra districts could be ascribed to intense anthropogenic activities (e.g., pigments, electroplating, and battery manufacturing). These industries have also been revealed to discharge effluents containing Pb into the aquatic environment [27].

#### 3.1.7. Iron (Fe)

In this study, the Fe concentrations reached the highest levels of 412.00 μg/L for monsoon and 303.29 μg/L for non-monsoon, with mean values of 117.35 and 145.35 μg/L, respectively. In the non-monsoon season, the maximum Fe values at several sites was close to the permissible limit. Generally, the elevated Fe concentrations in water samples implied that River Yamuna suffered from discharges of Fe-related industries in several areas such as Agra, in addition to various anthropogenic and geogenic causes.

#### 3.1.8. Zinc (Zn)

Zn level ranged between 4.93 and 279.27 μg/L for monsoon and 13.35 and 459.95 μg/L for non-monsoon, with mean values of 45.27 and 192.02 μg/L, respectively. Although the Zn levels in River Yamuna were higher than other heavy metals in all sites, it was within the safe limit (below 5000 μg/L) during both seasons(Fig 4B). Some studies reported possible risk to aquatic species and potential environmental risks of zinc in surface waters [33].

### 3.2. Correlation analysis for metals in water

Correlation analysis was performed for monsoon and non-monsoon seasons to assess the relationship among metals, as shown in S5 Table. Inter-metal interactions are indicative of metal sources and pathways in the media [34]. A positive correlation may indicate a common or similar source of these metals. In the present study, significant positive correlations were observed among various metal pairs: As-Cr, Cu-Cr, Pb-Cu, Pb-Ni, Fe-As, Fe-Cr, Fe-Cu, and Zn-Cu in monsoon, indicating a common source of origin. In the non-monsoon season, significant negative correlations were observed among metals pairs: Cr-Cd, Ni-Cr, and positive correlations were observed as Cu-Cd, Pb-Cd, Pb-Ni, Fe-As, Fe-Cr, Fe-Cu, and Zn-Pb. Fe shows a significant positive relationship with As, Cr, and Cu in both seasons. It is a naturally abundant metal and mainly comes from crustal sources. Chromium is used in leather, glass, and pigments industries.

### 3.3. Principal components analysis and cluster analysis for metals in water

PCA with Varimax normalized rotation was performed separately for monsoon and non-monsoon seasons to understand the relationships among the metals. Based on absolute loading values, the factor loadings were classified as ‘strong(>0.75)’, ‘moderate(0.75–0.50)’, and ‘weak’ (0.50–0.30) [35]. PCA yielded three PCs for the monsoon season and four PCs for the non-monsoon season with Eigenvalues >1, explaining 71.7% and 69.1% of the cumulative variance, respectively. PCA is depicted by loadings and score plot and is shown in S1 and S2 Figs for monsoon and non-monsoon, respectively. In the monsoon season, PC1, explaining 28.5% of total variance, had strong positive loadings (loadings>0.75)of As, Cr, and Fe (S1 Fig and S6 Table).

As is affected by natural factors, such as the parent rock composition [36] In addition to the natural source, effluents from thermal-based power plants also contribute to As contamination in river Yamuna [25]. The high As levels reported in this study could be ascribed to the increased industrial processes, automobile emissions, application of As-based pesticides, smelting of metals and usage of fossil fuels [37] at multiple districts such as Agra, Etawah, and Hamirpur. In general, Fe is present in relatively higher concentrations under natural conditions. Fe is a major component of crustal materials [38]. Cr is low in concentrations and is within the drinking water guideline limit. This component, therefore, appeared to be primarily associated with the geogenic source. Thus, metals in PC1, therefore, appeared to be primarily associated with the geogenic source. PC2 explained 24.8% of the total variance with strong loadings on Cu and Pb and moderate loading on Ni. These metals may have mainly come from anthropogenic sources such as industrial and urban discharges. Lead battery-based units are a common source of Ni and Pb [27]. In addition, these sites have high traffic density areas that would release toxic emissions containing Pb to the atmosphere, which are then deposited and accumulated into ecosystems. PC3 explaining 18.4% of total variance, showed strong loadings on Cd and Zn. Since the maximum concentrations of Cd and Zn in the water samples are within the WHO safe limit, it is inferred that this component represents a natural source. A similar result was reported in the surface water of the Lhasa River basin [39].

In the non-monsoon season, PC1 explained 18.3% showed strong positive loading on Cd and moderate loading on Cu (S2 Fig and S6 Table). Cd may have come from very unique anthropogenic sources such as from battery and dye-making industries [27]. In addition, Delhi and Mohana include large agricultural landscapes, utilizing pesticides and fertilizers that might release Cd into the aquatic environment. Cu may have its origin from both anthropogenic and natural sources. PC2 showed strong loading on Fe and moderate loading on Zn. Although both Fe and Zn are naturally present in the crust, their high concentrations in the river water during the dry season indicate that anthropogenic sources also contribute to them. This may include industrial and urban sewage discharges, electroplating industries, etc. Both As and Pb in PC3 that explained 16.7% of the total variance might have come from mixed sources in the non-monsoon season. Possible anthropogenic sources of As and Pb could be from industries and the use of agrochemicals.

In PC4, Ni showed strong positive loading and may have come from a unique anthropogenic source such as from some particular type of industrial discharges.

Hierarchical agglomerative cluster analysis was performed on the data set of both seasons using Ward’s linkage method using squared Euclidean distance. The result obtained for monsoon and the non-monsoon season is presented in the form of a dendrogram in S3 and S4 Figs. Three clusters are depicted in both seasons. In the first cluster representing monsoon season, As, Cr, Cu, and Ni are very well associated with each other (S3 Fig). The second cluster is comprised of Pb and Cd but is also linked to the first cluster. In the third cluster, Fe and Zn are very well linked with each other. Heavy metals such as As, Cr, Cu, and Ni during the monsoon season may be have come mainly from natural sources. Fe and Zn are, although are relatively high concentrations under natural conditions, may have come from anthropogenic sources as well, especially during the non-monsoon season. Pb and Cd may have come from mixed sources. In the non-monsoon season, Cr, As, Pb, Cu, and Ni in the first cluster may have come from mixed sources. Fe and Zn in the first cluster may have mainly natural origin (S4 Fig). Cd is separated from other groups indicating a unique source for this metal, and the result is similar to those of PCA. The main anthropogenic sources of various heavy metals in river Yamuna were linked to industrial sources (electroplating, dyeing, paper manufacturing, fertilizer, sugarcane etc) located on the banks of river, agricultural run-off, sewage discharge, agrochemical usage and vehicular sources [26–29].

### 3.4. Health risk assessment

Table 2A and 2B lists the statistical summary of the noncarcinogenic health risks in monsoon and non-monsoon season, respectively in terms of HQ and HI. In all districts, the mean HQ level of each metal was below 1 among adults in both monsoon and non-monsoon seasons. However, the mean HQ value estimated from As among children exceeded the safe limit of 1, suggesting that As was the main contributing element to non-cancer health risks of the River Yamuna. Similarly, Li and Zhang [40] demonstrated that As was the main pollutant that caused non carcinogenic risks to children, resulting in HI values above unity.

**Table 2:** a. Site-wise calculated values of HQ and HI for adults and children in monsoon season. b. Site-wise calculated values of HQ and HI for adults and children in non-monsoon season.

District	Group	As	Cd	Cr	Cu	Ni	Pb	Fe	Zn	HI
**a**										
Monsoon season										
**Poanta**	**Adult**	1.19×10^−01^	1.98×10^−02^	9.40×10^−06^	1.82×10^−03^	3.60×10^−03^	4.16×10^−03^	3.29×10^−03^	9.34×10^−03^	1.61×10^−01^
	**Children**	3.91×10^−01^	6.47×10^−02^	3.08×10^−05^	5.95×10^−03^	1.18×10^−02^	1.37×10^−02^	1.08×10^−02^	3.06×10^−02^	5.28×10^−01^
**Kalanaur**	**Adult**	9.66×10^−02^	1.17×10^−02^	4.95×10^−05^	2.89×10^−03^	4.40×10^−03^	4.75×10^−03^	5.09×10^−03^	8.71×10^−03^	1.34×10^−01^
	**Children**	3.16×10^−01^	3.82×10^−02^	1.62×10^−04^	9.44×10^−03^	1.44×10^−02^	1.55×10^−02^	1.67×10^−02^	2.85×10^−02^	4.39×10^−01^
**Mawi**	**Adult**	1.41×10^−01^	1.26×10^−02^	2.89×10^−05^	1.80×10^−03^	1.45×10^−02^	4.24×10^−03^	5.13×10^−03^	6.19×10^−04^	1.80×10^−01^
	**Children**	4.63×10^−01^	4.13×10^−02^	9.47×10^−05^	5.87×10^−03^	4.76×10^−02^	1.39×10^−02^	1.68×10^−02^	2.03×10^−03^	5.90×10^−01^
**Palla**	**Adult**	9.70×10^−02^	4.69×10^−02^	1.34×10^−04^	2.88×10^−03^	7.71×10^−03^	6.49×10^−03^	5.34×10^−03^	1.27×10^−03^	1.68×10^−01^
	**Children**	3.17×10^−01^	1.53×10^−01^	4.38×10^−04^	9.41×10^−03^	2.52×10^−02^	2.12×10^−02^	1.75×10^−02^	4.16×10^−03^	5.48×10^−01^
**Delhi**	**Adult**	2.54×10^−01^	3.27×10^−02^	1.05×10^−04^	1.79×10^−03^	6.39×10^−03^	4.14×10^−03^	5.05×10^−03^	6.03×10^−04^	3.05×10^−01^
	**Children**	8.31×10^−01^	1.07×10^−01^	3.44×10^−04^	5.85×10^−03^	2.09×10^−02^	1.35×10^−02^	1.65×10^−02^	1.97×10^−03^	9.97×10^−01^
**Mohana**	**Adult**	1.41×10^−01^	2.96×10^−02^	5.61×10^−05^	2.51×10^−03^	3.93×10^−03^	7.39×10^−03^	5.62×10^−03^	8.19×10^−04^	1.91×10^−01^
	**Children**	4.61×10^−01^	9.71×10^−02^	1.84×10^−04^	8.19×10^−03^	1.29×10^−02^	2.42×10^−02^	1.84×10^−02^	2.68×10^−03^	6.25×10^−01^
**Mathura**	**Adult**	2.73×10^−01^	1.29×10^−02^	4.65×10^−05^	2.08×10^−03^	4.40×10^−03^	4.63×10^−03^	4.93×10^−03^	8.23×10^−04^	3.02×10^−01^
	**Children**	8.92×10^−01^	4.24×10^−02^	1.52×10^−04^	6.83×10^−03^	1.44×10^−02^	1.51×10^−02^	1.61×10^−02^	2.69×10^−03^	9.90×10^−01^
**Agra**	**Adult**	7.59×10^−01^	1.64×10^−02^	2.59×10^−04^	1.39×10^−02^	1.45×10^−02^	2.61×10^−03^	5.03×10^−02^	3.41×10^−02^	8.92×10^−01^
	**Children**	2.49×10^+00^	5.38×10^−02^	8.46×10^−04^	4.55×10^−02^	4.75×10^−02^	8.55×10^−03^	1.65×10^−01^	1.12×10^−01^	2.92×10^+00^
**Etawah**	**Adult**	5.17×10^−01^	2.16×10^−02^	4.09×10^−04^	1.93×10^−02^	8.09×10^−03^	1.04×10^−02^	3.06×10^−02^	4.46×10^−03^	6.12×10^−01^
	**Children**	1.69×10^+00^	7.07×10^−02^	1.34×10^−03^	6.30×10^−02^	2.65×10^−02^	3.39×10^−02^	1.00×10^−01^	1.46×10^−02^	2.00×10^+00^
**Auraiya**	**Adult**	3.95×10^−01^	5.80×10^−03^	2.09×10^−04^	4.51×10^−03^	1.33×10^−02^	3.30×10^−03^	1.30×10^−02^	4.08×10^−03^	4.39×10^−01^
	**Children**	1.29×10^+00^	1.90×10^−02^	6.84×10^−04^	1.48×10^−02^	4.35×10^−02^	1.08×10^−02^	4.26×10^−02^	1.33×10^−02^	1.44×10^+00^
**Hamirpur**	**Adult**	5.25×10^−01^	6.62×10^−03^	4.47×10^−04^	4.81×10^−03^	2.01×10^−02^	5.51×10^−03^	2.30×10^−02^	3.04×10^−03^	5.88×10^−01^
	**Children**	1.72×10^+00^	2.17×10^−02^	1.46×10^−03^	1.57×10^−02^	6.58×10^−02^	1.80×10^−02^	7.52×10^−02^	9.95×10^−03^	1.93×10^+00^
**Rajapur**	**Adult**	4.80×10^−01^	6.43×10^−02^	2.39×10^−04^	5.43×10^−03^	6.41×10^−03^	1.27×10^−03^	1.69×10^−02^	1.20×10^−03^	5.75×10^−01^
	**Children**	1.57×10^+00^	2.10×10^−01^	7.82×10^−04^	1.77×10^−02^	2.10×10^−02^	4.16×10^−03^	5.54×10^−02^	3.94×10^−03^	1.88×10^+00^
**Pratappu**	**Adult**	2.60×10^−01^	5.28×10^−03^	3.76×10^−04^	5.54×10^−03^	6.78×10^−03^	2.44×10^−03^	1.81×10^−02^	2.83×10^−03^	3.01×10^−01^
	**Children**	8.51×10^−01^	1.73×10^−02^	1.23×10^−03^	1.81×10^−02^	2.22×10^−02^	7.99×10^−03^	5.93×10^−02^	9.27×10^−03^	9.86×10^−01^
**b**										
**Poanta**	**Adult**	1.09×10^−01^	2.71×10^−02^	5.94×10^−05^	4.98×10^−03^	6.14×10^−03^	3.22×10^−03^	5.95×10^−03^	1.59×10^−02^	1.72×10^−01^
	**Children**	3.57×10^−01^	8.86×10^−02^	1.94×10^−04^	1.63×10^−02^	2.01×10^−02^	8.43×10^−03^	7.27×10^−04^	1.95×10^−03^	4.93×10^−01^
**Kalanaur**	**Adult**	2.74×10^−01^	1.84×10^−02^	9.99×10^−05^	4.47×10^−03^	6.93×10^−03^	2.32×10^−03^	9.06×10^−03^	3.35×10^−02^	3.49×10^−01^
	**Children**	8.98×10^−01^	6.02×10^−02^	3.27×10^−04^	1.46×10^−02^	2.27×10^−02^	6.07×10^−03^	1.11×10^−03^	4.09×10^−03^	1.01×10^+00^
**Mawi**	**Adult**	3.52×10^−01^	8.04×10^−03^	1.07×10^−04^	2.49×10^−03^	7.86×10^−03^	3.95×10^−03^	5.82×10^−03^	8.93×10^−03^	3.89×10^−01^
	**Children**	1.15×10^+00^	2.64×10^−02^	3.49×10^−04^	8.16×10^−03^	2.57×10^−02^	1.04×10^−02^	7.12×10^−04^	1.09×10^−03^	1.22×10^+00^
**Palla**	**Adult**	2.59×10^−01^	2.67×10^−02^	7.44×10^−05^	6.61×10^−03^	9.96×10^−03^	5.47×10^−03^	8.70×10^−03^	7.07×10^−03^	3.24×10^−01^
	**Children**	8.49×10^−01^	8.75×10^−02^	2.44×10^−04^	2.16×10^−02^	3.26×10^−02^	1.43×10^−02^	1.06×10^−03^	8.64×10^−04^	1.01×10^+00^
**Delhi**	**Adult**	3.24×10^−01^	1.13×10^−01^	7.51×10^−05^	6.41×10^−03^	2.33×10^−02^	1.70×10^−02^	3.71×10^−02^	1.09×10^−02^	5.31×10^−01^
	**Children**	1.06×10^+00^	3.71×10^−01^	2.46×10^−04^	2.10×10^−02^	7.62×10^−02^	4.46×10^−02^	4.53×10^−03^	1.34×10^−03^	1.58×10^+00^
**Mohana**	**Adult**	3.66×10^−01^	1.30×10^−01^	1.05×10^−04^	4.11×10^−03^	1.32×10^−02^	1.78×10^−02^	3.20×10^−02^	9.96×10^−03^	5.73×10^−01^
	**Children**	1.20×10^+00^	4.26×10^−01^	3.45×10^−04^	1.34×10^−02^	4.30×10^−02^	4.66×10^−02^	3.91×10^−03^	1.22×10^−03^	1.73×10^+00^
**Mathura**	**Adult**	5.06×10^−01^	1.04×10^−01^	8.29×10^−05^	8.58×10^−03^	1.22×10^−02^	1.24×10^−02^	1.05×10^−02^	3.94×10^−02^	6.94×10^−01^
	**Children**	1.66×10^+00^	3.42×10^−01^	2.71×10^−04^	2.81×10^−02^	3.98×10^−02^	3.24×10^−02^	1.29×10^−03^	4.82×10^−03^	2.11×10^+00^
**Agra**	**Adult**	4.33×10^−01^	5.21×10^−02^	2.52×10^−04^	8.64×10^−03^	9.30×10^−03^	1.50×10^−02^	3.39×10^−02^	5.62×10^−02^	6.08×10^−01^
	**Children**	1.42×10^+00^	1.71×10^−01^	8.24×10^−04^	2.83×10^−02^	3.05×10^−02^	3.93×10^−02^	4.15×10^−03^	6.87×10^−03^	1.70×10^+00^
**Etawah**	**Adult**	5.50×10^−01^	2.56×10^−02^	1.82×10^−04^	6.42×10^−03^	1.00×10^−02^	7.58×10^−03^	2.41×10^−02^	3.11×10^−02^	6.55×10^−01^
	**Children**	1.80×10^+00^	8.37×10^−02^	5.97×10^−04^	2.10×10^−02^	3.28×10^−02^	1.98×10^−02^	2.94×10^−03^	3.80×10^−03^	1.97×10^+00^
**Auraiya**	**Adult**	5.57×10^−01^	1.98×10^−02^	1.53×10^−04^	6.96×10^−03^	7.06×10^−03^	6.64×10^−03^	2.48×10^−02^	2.44×10^−02^	6.47×10^−01^
	**Children**	1.82×10^+00^	6.47×10^−02^	4.99×10^−04^	2.27×10^−02^	2.31×10^−02^	1.74×10^−02^	3.04×10^−03^	2.99×10^−03^	1.96×10^+00^
**Hamirpur**	**Adult**	5.70×10^−01^	2.43×10^−02^	1.46×10^−04^	7.74×10^−03^	9.84×10^−03^	7.18×10^−03^	1.83×10^−02^	3.53×10^−02^	6.72×10^−01^
	**Children**	1.86×10^+00^	7.96×10^−02^	4.79×10^−04^	2.53×10^−02^	3.22×10^−02^	1.88×10^−02^	2.23×10^−03^	4.32×10^−03^	2.03×10^+00^
**Rajapur**	**Adult**	5.38×10^−01^	1.02×10^−02^	8.41×10^−05^	3.97×10^−03^	7.46×10^−03^	4.13×10^−03^	9.99×10^−03^	1.63×10^−03^	5.75×10^−01^
	**Children**	1.76×10^+00^	3.34×10^−02^	2.75×10^−04^	1.30×10^−02^	2.44×10^−02^	1.08×10^−02^	1.22×10^−03^	1.99×10^−04^	1.84×10^+00^
**Pratappu**	**Adult**	2.97×10^−01^	3.19×10^−02^	2.05×10^−04^	7.74×10^−03^	5.64×10^−03^	6.72×10^−03^	1.07×10^−02^	3.07×10^−02^	3.91×10^−01^
	**Children**	9.73×10^−01^	1.05×10^−01^	6.72×10^−04^	2.53×10^−02^	1.85×10^−02^	1.76×10^−02^	1.30×10^−03^	3.75×10^−03^	1.14×10^+00^

The data in Table 2A and 2B also depicted that the HI values exceeded the safe limit among children during both seasons, implying that children’s exposure to the water source could potentially trigger adverse non-cancer health effects. Children have higher occasions to interact with environmental contaminants than adults because of their behavioral and physical activities, playing periods, and inattention during eating and drinking food items [40]. Overall, in the monsoon season, the HI values at different sites followed the order of Agra >Etawah> Hamirpur >Rajapur>Auraiya> Delhi Rly Bridge > Mathura >Pratappur> Mohana >Mawi>Palla>Poanta>Kalanaur. In the non-monsoon season, the HI values at different sites followed the order of Mathura>Hamirpur>Etawah>Auraiya>Rajapur>Mohana>Agra>Delhi Rly Bridge >Mawi>Pratappur>Kalanaur>Palla>Poanta.

Although the HI data provided an appropriate indication of the noncarcinogenic health effects for residents, these values might suffer from a degree of uncertainty [3]. For instance, the exposure parameters and water consumption rates used to characterize the risks might vary according to regional or individual differences. FHI is presented in the following section based on local and real experience for drinking water contamination to minimize the uncertainty sources and sustain a healthy aquatic environment.

### 3.5. Fuzzy-based HI classification

#### 3.5.1. Fuzzy hazard index (FHI) results

**Fig 5:**
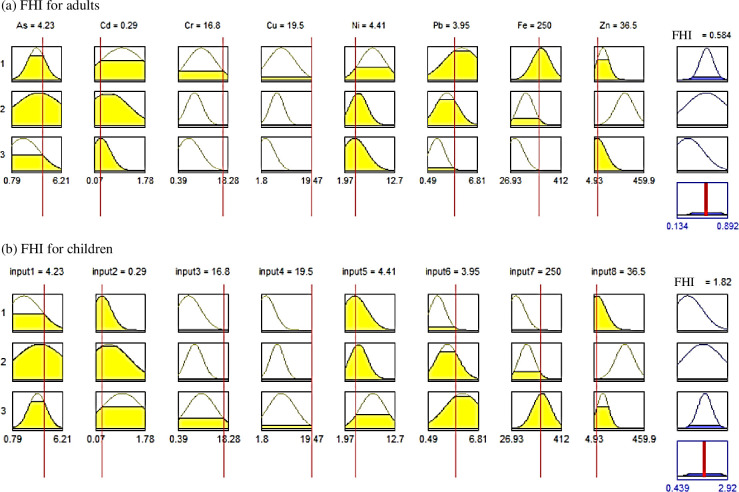
Rule Viewer showing the roadmap of the whole fuzzy inference process for estimating FHI in the Yamuna river for (a) adults, and (b) children.

One of the major problems of the health risk assessment is the uncertainty in the HI values because the employed parameters (e.g., intake rate of water and body weight) can differ among districts and locations. Ali Hosseini et al. [41] suggested that a new “Quality Index” hypothesis could be established based on the fuzzy logic theory to deal with ambiguous and biased concepts and data. The estimated values of FHI, based on practical experience and understanding of the environmental conditions of the Yamuna River, are shown in the fuzzy inference diagram (Fig 5). The nine plots across the top of this structure represent the antecedent and consequent of the first rule. The first eight columns of plots (the 24 yellow plots) denote the membership functions referenced by the antecedent, or the “If” part of each rule (S3 and S4 Tables). Rule 1 crisply maps cluster 1 in the input space to cluster 1 in the output space. Similarly, the other two rules map cluster 2 and cluster 3 in the input space to cluster 2 and cluster 3 in the output space, respectively. The ninth column of plots (the three blue plots) demonstrates the membership functions referenced by the consequent or the “Then” part of each rule. The fourth plot in the ninth column represents the aggregate weighted decision for the entire inference system. The defuzzified decision (crisp output) is shown as a bold vertical line on this plot. The metal variables and their instant concentrations are positioned at the top of the columns.

For the case of As, Cd, Cr, Cu, Ni, Pb, Fe, and Zn, concentrations of 4.23, 0.29, 16.75, 19.47, 4.41, 3.95, 250.33, and 36.47 μg/L, respectively, the estimated FHIs are 0.58 for adults (Fig 5A) and 1.82 for children (Fig 5B). These defuzzified outputs corresponded to the estimated HIs of 0.61 and 2.00, respectively, equivalent to the aggregation of about 80% out1cluster 1, 10% out1cluster 2, and 10% out1cluster3. The degree of membership allowed for a reliable understanding and practical meaning of the hazard index, especially for public concerns. Hence, the fuzzy theory was employed to overcome the traditional fact that a little shift in metal concentration around its permissible limit would totally change the degree of health risk.

A similar observation has been reported by Tiri et al. [22], who estimated a water quality index of Oued El-Hai Basin based on fuzzy logic, using ten parameters (e.g., pH, TDS, Ca, Mg, Na, K). The fuzzy water quality index (FWQI) results were compared with the actual WQI, resulting in a correlation coefficient within the 0.88–0.99 range [22].

#### 3.5.2. Fuzzy model applicability

**Fig 6:**
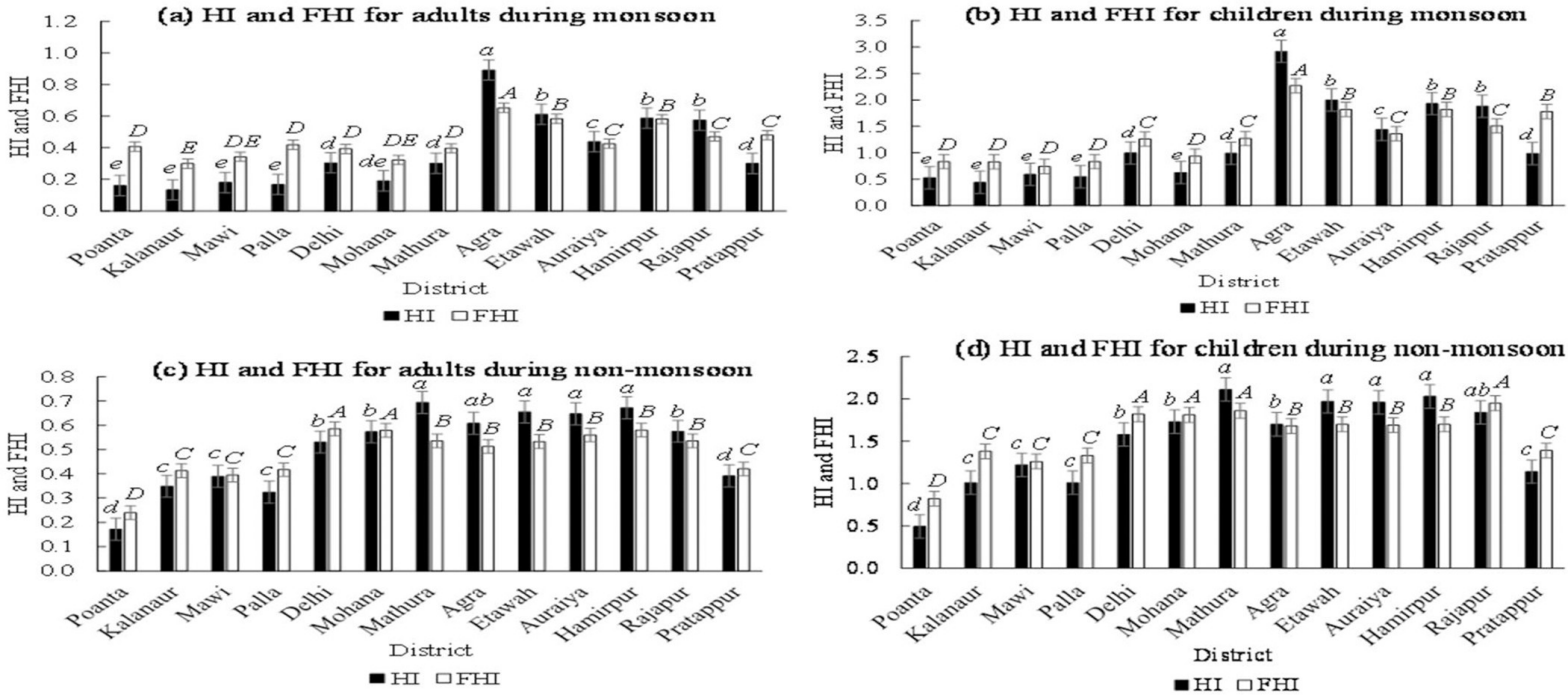
Comparison between new FHI and traditional HI estimated at 13 districts of the river Yamuna: (a) adults during monsoon season, (b) children during monsoon season, (c) adults during non-monsoon season, and (d) children during non-monsoon season. Groups sharing the same letter do not statistically differ from each other at the specified significance level (alpha = 0.05).

The fuzzy model results were compared with the actual HI data estimated by Eq 3, at the 13 districts of the Yamuna stretch (Fig 6A–6D). One-way ANOVA was applied to analyze the significant differences among sampling stations for health risks in terms of HI and FHI. Tukey’s *t*-test was also performed to identify the homogeneous type of the data sets. HI and FHI values showed significant difference (p < 0.05) among sampling locations in both monsoon and non-monsoon seasons. The HI values during the non-monsoon season for children ranged between 0.49 and 2.11, with an average value of 1.52 ± 0.50. These indices correspond to FHIs of 0.82 (minimum) and 1.95 (maximum), with an average value of 1.57 ± 0.32. The adequate correlation between the HI and FHI values (*R*^2^: 0.75–0.83) revealed the effectiveness and reliability of the fuzzy logic tool in predicting the noncarcinogenic hazard indices depending on the human expert knowledge and experience. Moreover, the results of both HI and FHI depicted that children were subjected to higher risks due to metals exposure as compared with adults. Also, both HI and FHI retained their maximum values at Agra, Etawah, and Hamirpur (Fig 6A–6D). Additionally, the classic HQ and/or HI values were estimated from exposure to a specific source (river); however, this was not the case for the FHI approach that used the human understanding of the multiple pollution pathways in the study area. These benefits also ensure that human expert knowledge is essential in determining the type of water treatment required to meet national and international standards. In addition, the fuzzy logic concept should be incorporated into the national water quality monitoring program to establish a consolidated framework for managing the river systems.

Mohanta et al. [3] proposed an index model using the fuzzy logic approach to investigate the effect of fluoride on human health (adults and children). Fluoride concentration and FHI were the model’s input and output, respectively. The study revealed a high determination coefficient between FHI and HI, reaching up to *R*^2^ = 0.9755. Moreover, the study demonstrated that the FHI approach attained more concise, stringent, and consistent results compared with the conventional HI method. Hence, FHI could include both qualitative and quantitative variables with different values and meanings to estimate the hazard index associated with adults and children during the monsoon and non-monsoon seasons.

Mohanta and Mishra [23] used the fuzzy theory to develop cancer risk (FCR) and hazard index (FHI) associated with men, women, and children for aniline-enriched groundwater. The model’s input (antecedent) was the aniline concentration described by triangular and trapezoidal membership functions. The results of FCR and FHI were positively correlated with the data estimated from the conventional USEPA method, with *R*^2^ values of 0.97 and 0.99, respectively. The sufficient *R*^2^ for validation implied that fuzzy logic would highly predict risks associated with human health.

Li et al. [12] proposed a fuzzy water pollution index (FWPI) to assess the quality of Qu River based on 125 fuzzy If-Then rules. The model’s inputs (antecedent sets) were DO, COD, BOD, NH_3_-N, and TP, incorporated as trapezoid and triangular membership functions. Their study demonstrated that the results of FWPI were consistent with those of fuzzy comprehensive evaluation and grey relational model methods. Similar findings were observed by Icaga [42], who proposed an index model for water quality evaluation using fuzzy logic with eleven water quality parameters (as inputs). Their study demonstrated that environmentalists should be well experienced in giving correct and precise field survey responses, leading to satisfactory fuzzy model accuracy.

## 4. Conclusion

The study revealed that higher levels of most of the metals in the non-monsoon season in the river Yamuna could be attributed to low flow conditions and less dilution. Overall, natural and anthropogenic sources contributed to the heavy metals in Yamuna river water. Major anthropogenic sources may include industrial and urban discharges and the use of agrochemicals. An earlier study also indicated that the river was affected by pollution coming from untreated household sewage, industrial effluents, and fertilizers used in agriculture [2]. Health risk assessment indicated possible health threats to the children (HI>1 and As was the main contributing element to non-cancer risk in both the seasons in most sampled sites. Fuzzy logic-based health risk assessment also indicated that children’s exposure to the Yamuna river water might potentially trigger adverse non-cancer health effects. The study further elucidated that incorporating fuzzy logic concept in water quality monitoring may contribute to developing a consolidated agenda for managing the river ecosystems.

## Supporting information

S1 Table Geo-environmental conditions of river Yamuna. (DOC)

S2 Table Thirteen monitoring stations distributed along Yamuna River for trace element measurement. (DOC)

S3 Table Fuzzy membership functions of input and output variables for estimating FHI among adults. (DOC)

S4 Table Fuzzy membership functions of input and output variables for estimating FHI among children. (DOC)

S5 Table Correlation of metals in river Yamuna in monsoon and non-monsoon season. (DOC)

S6 Table Factor loading for monsoon and non-monsoon seasons, respectively. (DOC)

S1 Fig PCA loadings and score plots of heavy metals in monsoon season. (TIF)

S2 Fig PCA loadings and score plots of heavy metals in the non-monsoon season. (TIF)

S3 Fig Dendrogram showing clustering of heavy metals in water for monsoon season. (TIF)

S4 Fig Dendrogram showing clustering of heavy metals in water for non-monsoon season. (TIF)

## References

[1] Parmar K, Bhardwaj R. Statistical, time series, and fractal analysis of full stretch of river Yamuna (India) for water quality management. Enviro Sci Pollut Res. 2015; 22: 397–414. 10.1007/s11356-014-3346-1 25077654

[2] Jaiswal M, Hussain J, Gupta SK, Nasr M, Nema AK. Comprehensive evaluation of water quality status for the entire stretch of Yamuna River, India. Environ Monit Assess. 2019; 191: 208. doi: 10.1007/s10661-019-7312-8 30847649

[3] Mohanta VL, Singh S, Mishra BK. Human health risk assessment of fluoride-rich groundwater using fuzzy-analytical process over the conventional technique. Groundwater Sustain Devel. 2020; 10: 100291. 10.1016/j.gsd.2019.100291

[4] Valdés J, Guinez M, Castillo A, Vega SE. Cu, Pb, and Zn content in sediments and benthic organisms from San Jorge Bay (northern Chile): accumulation and biotransference in subtidal coastal systems. Cienc Marinas. 2014; 40:45–58. 10.7773/cm.v40i1.2318.

[5] Tchounwou PB, Yedjou CG, Patlolla AK, Sutton DJ. Heavy metal toxicity and the environment. Mol Clin Environ Toxicol. 2012; 101: 133–164. doi: 10.1007/978-3-7643-8340-4_6 22945569 PMC4144270

[6] Balali-Mood M, Naseri K, Tahergorabi Z, Khazdair MR, Sadeghi M. Toxic mechanisms of five heavy metals: Mercury, Lead, Chromium, Cadmium, and Arsenic. Front Pharmacol. 2021; 12:643972. doi: 10.3389/fphar.2021.643972 33927623 PMC8078867

[7] Sall ML, Diaw AKD, Gningue-Sall D, Aaron SE, Aaron J-J. Toxic heavy metals: impact on the environment and human health, and treatment with conducting organic polymers, a review. Environ Sci Pollut Res. 2020. 27:29927–29942. 10.1007/s11356-020-09354-3 32506411

[8] Odobašic´ A, Šestan I, Begic´ S. Biosensors for determination of heavy metals in waters. Biosensors Environmental Monitoring. IntechOpen. 2019. 10.5772/intechopen.84139

[9] Suthar S, Nema AK, Chabukdhara M, Gupta SK. Assessment of metals in water and sediments of Hindon River, India: Impact of industrial and urban discharges. J Hazardous Mat. 2009; 171: 1088–1095. doi: 10.1016/j.jhazmat.2009.06.109 19616893

[10] Gupta S.K., Chabukdhara M., Pandey P.K., Singh J., Bux F. (2014). Evaluation of potential ecological risk of metal contamination in the Gomti River: a biomonitoring approach. Ecotox Environ Saf. 110, 49–55. doi: 10.1016/j.ecoenv.2014.08.008 25194696

[11] Chabukdhara M, Gupta SK, Nema AK. Assessment of seasonal variation of surface water quality using environmetric and indexing approach. IIOAB Journal, special issue "Water, Air and Soil Pollution: Monitoring and remediation. " IIOAB Journal. 2016; 7: 16–24.

[12] Li R., Zou Z., & An Y. (2016) Water quality assessment in Qu River based on fuzzy water pollution index method. Journal of Environmental Sciences (China), 50, 87–92. doi: 10.1016/j.jes.2016.03.030 28034435

[13] Githaiga KB, Njuguna SM, Gituru RW, Yan X. Water quality assessment, multivariate analysis and human health risks of heavy metals in eight major lakes in Kenya. J Env Manage. 2021; 297: 113410. 10.1016/j.jenvman.2021.113410 34346396

[14] Sharma D, Kansal A. Water quality analysis of River Yamuna using water quality index in the national capital territory, India (2000–2009). Appl Water Sci. 2011; 1, 147–157. 10.1007/s13201-011-0011-4

[15] APHA. Standard methods for the examination of water and wastewater (21th Ed.). Washington, D.C.: American Public Health Association; 2010.

[16] Nasr M, Zahran H. Performance evaluation of agricultural drainage water using modeling and statistical approaches. Egypt J Aquat Res. 2016; 42: 141–148. 10.1016/j.ejar.2016.04.006

[17] Chabukdhara M., Nema A.K., 2012. Assessment of heavy metal contamination in Hindon River sediments: a chemometric and geochemical approach. Chemosphere; 87: 945–953. doi: 10.1016/j.chemosphere.2012.01.055 22406241

[18] USEPA (US Environmental Protection Agency). Definitions and general principles for exposure assessment. Guidelines for exposure assessment. Washington, D.C. Office of Pesticide Programs, USA. 1992.

[19] USEPA (US Environmental Protection Agency). Risk Assessment Guidance for Superfund, Vol 1. Human Health Evaluation Manual. Part A (interim final), EPA/540/1-89/002. Office of Emergency and Remedial Response, Washington, DC, USA. 1989.

[20] Zadeh LA. Fuzzy sets. Inf Control.1965; 8(3):338–353. 10.1016/S0019-9958(65)90241-X

[21] Gupta SK, Ansari FA, Nasr M, Rawat I, Nayunigari MK, Bux F. Cultivation of Chlorella sorokiniana and Scenedesmus obliquus in wastewater: Fuzzy intelligence for evaluation of growth parameters and metabolites extraction. J Cleaner Prod; 2017, 147:419–430.

[22] Tiri A, Belkhiri L, Mouni L. Evaluation of surface water quality for drinking purposes using fuzzy inference system. Groundwater Sustain Develop. 2018; 6:235–244. 10.1016/j.gsd.2018.01.006

[23] Mohanta VL, Mishra BK. Integration of cancer and non-cancer human health risk assessment for Aniline enriched groundwater: a fuzzy inference system-based approach. Environ Geochem Heal. 2020; 42: 3623–3639. doi: 10.1007/s10653-020-00590-7 32419090

[24] Kumar M, Sharif M, Ahmed S. Impact of urbanization on the river Yamuna basin. Int. J. River Basin Manage. 2020;18: 461–475. 10.1080/15715124.2019.1613412

[25] Dubey CS, Mishra BK, Shukla DP, Singh RP, Tajbakhsh M, Sakhare P. Anthropogenic arsenic menace in Delhi Yamuna Flood Plains. Environ Earth Sci. 2012; 65:131–139. 10.1007/s12665-011-1072-2

[26] Sehgal M, Garg A, Suresh R, Dagar P. Heavy metal contamination in the Delhi segment of Yamuna basin. Environ Monit Assess. 2012; 184:1181–1196. doi: 10.1007/s10661-011-2031-9 21505769

[27] Bhardwaj R, Gupta A, Garg JK. Evaluation of heavy metal contamination using environ-metrics and indexing approach for River Yamuna, Delhi stretch, India. Water Sci. 2017; 31: 52–66. 10.1016/j.wsj.2017.02.002

[28] Kaushik A, Kansal A, Meena S, Kumari S, Kaushik C.P Heavy metal contamination of river Yamuna, Haryana, India: Assessment by Metal Enrichment Factor of the Sediments. J Hazard Mat. 2009; 164: 265–270. 10.1016/j.jhazmat.2008.08.031 18809251

[29] Asim M, Rao KN. Assessment of heavy metal pollution in Yamuna River, Delhi-NCR, using heavy metal pollution index and GIS. Environ Monit Assess. 2021; 193: 103. doi: 10.1007/s10661-021-08886-6 33517501

[30] BIS. Bureau of Indian Standard Specification for Drinking Water. IS: 10500: 2012. (Second Revision). 2012; BIS, New Delhi.

[31] WHO. Guidelines for Drinking-water Quality, fourth ed. World Health Organization, Geneva, Switzerland, 2011. http://apps.who.int/iris/bitstream/10665/44584/1/9789241548151_eng.pdf.

[32] Hussain J, Husain I, Arif M. et al. Studies on heavy metal contamination in Godavari river basin. Appl Water Sci. 2017; 7, 4539–4548. 10.1007/s13201-017-0607-4

[33] Andarani P, Yokota K, Saga M, Inoue T, Matsumoto Y. Study of zinc pollution in river water: Average mass balance based on irrigation schedule. River Res Applic. 2020; 36: 1286– 1295. 10.1002/rra.3632

[34] Chabukdhara M, Gupta SK, Kotecha Y, Nema AK. Groundwater quality in Ghaziabad district, India: multivariate and health risk assessment. Chemosphere, 2017; 179:167–178. doi: 10.1016/j.chemosphere.2017.03.086 28365502

[35] Liu CW, Lin KH, Kuo YM. Application of factor analysis in the assessment of groundwater quality in a Blackfoot disease area in Taiwan. Sci Total Environ. 2003; 313: 77–89. doi: 10.1016/S0048-9697(02)00683-6 12922062

[36] Salati S, Moore F. Assessment of heavy metal concentration in the Khoshk River water and sediment, Shiraz, Southwest Iran. Environ Monit Assess. 2010; 164:677–689. doi: 10.1007/s10661-009-0920-y 19421887

[37] Osuna-Martínez CC, Armienta MA, Bergés-Tiznado ME, Páez-Osuna F. Arsenic in waters, soils, sediments, and biota from Mexico: An environmental review. Sci Tot Environ. 2021; 752:142062. 10.1016/j.scitotenv.2020.142062 33207489

[38] Kumar AV, Patil RS, Nambi KSV. Source apportionment of suspended particulate matter at two traffic junctions in Mumbai, India. Atmos Environ. 2001; 35:4245–4251. 10.1016/S1352-2310(01)00258-8

[39] Mao G., Zhao Y., Zhang F., Liu J., Huang X. Spatiotemporal variability of heavy metals and identification of potential source tracers in the surface water of the Lhasa River basin. Environ Sci Poll Res. 2019; 26: 7442–7452. doi: 10.1007/s11356-019-04188-0 30694435

[40] Li Siyue, Zhang Quanfa. Risk assessment and seasonal variations of dissolved trace elements and heavy metals in the Upper Han River, China. J. Hazard. Mater. 2010; 181:1051–1058. doi: 10.1016/j.jhazmat.2010.05.120 20638969

[41] Ali Hosseini S, Shirani AS, Lotfi M, Menhaj MB. Design and application of supervisory control based on neural network PID controllers for pressurizer system, Prog Nucl Ener. 2020; 130;103570. 10.1016/j.pnucene.2020.103570

[42] Icaga Y. Fuzzy evaluation of water quality classification. Ecol Ind. 2007; 7:710–718. 10.1016/j.ecolind.2006.08.002

