# Supplementary material for: Heavy metal contamination in the complete stretch of Yamuna river: A fuzzy logic approach for comprehensive health risk assessment
Source: PLoS One. 2022 Aug 8;17(8):e0272562. doi: 10.1371/journal.pone.0272562 (PMC9359575; doi:10.1371/journal.pone.0272562)
Supplement: S1 Table — (DOC) [file pone.0272562.s001.doc]

**Table S1.** Geo-environmental conditions of River Yamuna.

| Parameters | Details | Reference |
| --- | --- | --- |
| Origin | Yamunotri Glacier near Banderpoonch peaks (30º59′ N and 78º27′ E) in the Mussourie range, lower Himalayas | http://uyrb.gov.in/ |
| Elevation | 6,387 m above mean sea level | http://uyrb.gov.in/ |
| River traveling distance | About 1,376 kilometers | http://uyrb.gov.in/ |
| Important tributaries | Chambal, Betwa, Sind, Ken, and Mandakini | http://uyrb.gov.in/ |
| Catchment area | 366,223 km^2^ | http://uyrb.gov.in/ |
| Catchment basin area | 345,848 km^2^ | http://uyrb.gov.in/ |
| Water spread area | 20,375 km^2^ | http://uyrb.gov.in/ |
| Average annual rainfall | 400 and 1500 mm | (CPCB, 2006) |
| Annual mean maximum temperature | Varies between 24.0 and 42.5°C | (CPCB, 2006) |
| Annual mean minimum temperature | Varies between − 1.0 and 11.0°C in the basin area | (CPCB, 2006) |
| Topography of the Yamuna River | Hilly foothills and plateau region, plains, and valleys | (CPCB, 2006) |
| Soil types | Alluvial (42%), medium black (25.5%), and mixed red and black type (15%), | (CPCB, 2006) |
| Major land-use types in the river basin | Agriculture (60%), forest (12.5%), urban and barren land (2.9%), non-arable 24.6% | (CPCB, 2006; Rai et al., 2012; Sharma et al., 2020). |
