# Supplementary material for: Heavy metal contamination in the complete stretch of Yamuna river: A fuzzy logic approach for comprehensive health risk assessment
Source: PLoS One. 2022 Aug 8;17(8):e0272562. doi: 10.1371/journal.pone.0272562 (PMC9359575; doi:10.1371/journal.pone.0272562)
Supplement: S2 Table — (DOC) [file pone.0272562.s002.doc]

**Table S2.** Thirteen monitoring stations distributed along Yamuna River for trace element measurement.

| Site | Catchment area (km^2^) | Latitude | Longitude | Location and State |
| --- | --- | --- | --- | --- |
| Paonta | 10769 | 30°25'31'' | 77°35'31'' | Sirmaur, Himachal Pradesh |
| Kalanaur | 12639 | 30°04'10'' | 77°21'52'' | Saharanpur, Uttar Pradesh |
| Mawi | 15622 | 29°23'07'' | 77°09'16'' | Muzaffarnagar, Uttar Pradesh |
| Palla | 17324 | 28°49'46'' | 77°13'27'' | North West Delhi, Delhi |
| Delhi | 18552 | 28°39'45'' | 77°14'48'' | North Delhi, Delhi |
| Mohana | 27670 | 28°14'58'' | 77°28'12'' | Faridabad, Haryana |
| Mathura | 47463 | 27°26'30'' | 77°42'54'' | Mathura, Uttar Pradesh |
| Agra | 49052 | 27°15'15'' | 78°01'23'' | Agra, Uttar Pradesh |
| Etawah | 98715 | 26°45'00'' | 78°59'00'' | Etawah, Uttar Pradesh |
| Auraiya | 261331 | 26°25'34'' | 79°25'00'' | Auraiya, Uttar Pradesh |
| Hamirpur | 276789 | 25°57'39'' | 80°09'16'' | Hamirpur, Uttar Pradesh |
| Rajapur | 364552 | 25°23'23" | 81°09'15" | Chitrakoot, Uttar Pradesh |
| Pratappur | 366522 | 25°21'17'' | 81°40'02'' | Allahabad, Uttar Pradesh |
