# Supplementary material for: Heavy metal contamination in the complete stretch of Yamuna river: A fuzzy logic approach for comprehensive health risk assessment
Source: PLoS One. 2022 Aug 8;17(8):e0272562. doi: 10.1371/journal.pone.0272562 (PMC9359575; doi:10.1371/journal.pone.0272562)
Supplement: S3 Table — (DOC) [file pone.0272562.s003.doc]

**Table S3.** Fuzzy membership functions of input and output variables for estimating FHI among adults.

| Label | Description | Gaussian shape membership function | |
| --- | --- | --- | --- |
|  |  | Standard deviation and mean | Range |
| in1cluster1 | The first membership function associated with input 1 | 0.955 and 3.536 | [0.79 – 6.21] |
| in1cluster2 | The second membership function associated with input 1 | 2.645 and 3.783 | [0.79 – 6.21] |
| in1cluster3 | The third membership function associated with input 1 | 1.784 and 2.076 | [0.79 – 6.21] |
| in2cluster1 | The first membership function associated with input 2 | 0.714 and 1.009 | [0.07 – 1.78] |
| in2cluster2 | The second membership function associated with input 2 | 0.695 and 0.546 | [0.07 – 1.78] |
| in2cluster3 | The third membership function associated with input 2 | 0.327 and 0.296 | [0.07 – 1.78] |
| in3cluster1 | The first membership function associated with input 3 | 4.985 and 8.817 | [0.39 – 18.28] |
| in3cluster2 | The second membership function associated with input 3 | 2.940 and 6.380 | [0.39 – 18.28] |
| in3cluster3 | The third membership function associated with input 3 | 4.391 and 4.253 | [0.39 – 18.28] |
| in4cluster1 | The first membership function associated with input 4 | 4.750 and 8.998 | [1.80 – 19.47] |
| in4cluster2 | The second membership function associated with input 4 | 2.418 and 7.454 | [1.80 – 19.47] |
| in4cluster3 | The third membership function associated with input 4 | 3.165 and 3.427 | [1.80 – 19.47] |
| in5cluster1 | The first membership function associated with input 5 | 2.637 and 8.046 | [1.97 – 12.70] |
| in5cluster2 | The second membership function associated with input 5 | 1.912 and 4.987 | [1.97 – 12.70] |
| in5cluster3 | The third membership function associated with input 5 | 2.678 and 4.150 | [1.97 12.70] |
| in6cluster1 | The first membership function associated with input 6 | 2.248 and 4.886 | [0.49 – 6.81] |
| in6cluster2 | The second membership function associated with input 6 | 1.392 and 2.971 | [0.49 – 6.81] |
| in6cluster3 | The third membership function associated with input 6 | 0.985 and 1.729 | [0.49 – 6.81] |
| in7cluster1 | The first membership function associated with input 7 | 69.96 and 264.10 | [26.93 – 412.00] |
| in7cluster2 | The second membership function associated with input 7 | 57.84 and 147.50 | [26.93 – 412.00] |
| in7cluster3 | The third membership function associated with input 7 | 67.41 and 63.61 | [26.93 – 412.00] |
| in8cluster1 | The first membership function associated with input 8 | 53.62 and 89.01 | [4.93 – 459.90] |
| in8cluster2 | The second membership function associated with input 8 | 82.9 and 289.1 | [4.93 – 459.90] |
| in8cluster3 | The third membership function associated with input 8 | 81.38 and 36.32 | [4.93 – 459.90] |
| out1cluster1 | The first membership function associated with output | 0.0985 and 0.5860 | [0.134 – 0.892] |
| out1cluster2 | The second membership function associated with output | 0.3072 and 0.5801 | [0.134 – 0.892] |
| out1cluster3 | The third membership function associated with output | 0.2311 and 0.3031 | [0.134 – 0.892] |
