# Supplementary material for: Heavy metal contamination in the complete stretch of Yamuna river: A fuzzy logic approach for comprehensive health risk assessment
Source: PLoS One. 2022 Aug 8;17(8):e0272562. doi: 10.1371/journal.pone.0272562 (PMC9359575; doi:10.1371/journal.pone.0272562)
Supplement: S5 Table — (DOC) [file pone.0272562.s005.doc]

**Table S5.** Correlation of metals in river Yamuna in monsoon and non-monsoon season

| **Metals** | **As** | **Cd** | **Cr** | **Cu** | **Ni** | **Pb** | **Fe** | **Zn** |
| --- | --- | --- | --- | --- | --- | --- | --- | --- |
| **Monsoon** |  |  |  |  |  |  |  |  |
| As | 1 |  |  |  |  |  |  |  |
| Cd | 0.15 | 1 |  |  |  |  |  |  |
| Cr | 0.46^**^ | -0.15 | 1 |  |  |  |  |  |
| Cu | 0.26 | -0.17 | 0.50^**^ | 1 |  |  |  |  |
| Ni | 0.21 | -0.07 | 0.25 | 0.28 | 1 |  |  |  |
| Pb | -0.13 | 0.05 | 0.12 | 0.38^*^ | 0.34^*^ | 1 |  |  |
| Fe | 0.65^**^ | 0.19 | 0.72^**^ | 0.51^**^ | 0.24 | 0.09 | 1 |  |
| Zn | 0.28 | 0.29 | 0.18 | 0.53^**^ | 0.05 | 0.07 | 0.53 | 1 |
| **Non-Monsoon** |  |  |  |  |  |  |  |  |
| As | 1 |  |  |  |  |  |  |  |
| Cd | 0.11 | 1 |  |  |  |  |  |  |
| Cr | 0.11 | -0.24^**^ | 1 |  |  |  |  |  |
| Cu | 0.05 | 0.25^**^ | 0.04 | 1 |  |  |  |  |
| Ni | -0.01 | 0.06 | -0.17^*^ | -0.06 | 1 |  |  |  |
| Pb | 0.13 | 0.3^**^ | -0.11 | 0.01 | 0.25^**^ | 1 |  |  |
| Fe | 0.24^**^ | 0.01 | 0.19^*^ | 0.19^*^ | 0.03 | 0.08 | 1 |  |
| Zn | 0.05 | 0.02 | 0.07 | 0.14 | 0.07 | 0.27^**^ | 0.13 | 1 |

^**^Correlation is significant at the 0.01 level (2-tailed)

^*^Correlation is significant at the 0.05 level (2-tailed)
