# Supplementary material for: Heavy metal contamination in the complete stretch of Yamuna river: A fuzzy logic approach for comprehensive health risk assessment
Source: PLoS One. 2022 Aug 8;17(8):e0272562. doi: 10.1371/journal.pone.0272562 (PMC9359575; doi:10.1371/journal.pone.0272562)
Supplement: S6 Table — (DOC) [file pone.0272562.s006.doc]

**Table S6.** Factor loading for monsoon and non-monsoon seasons, respectively

| Variables | Monsoon | | | Non-Monsoon | | | |
| --- | --- | --- | --- | --- | --- | --- | --- |
|  | PC1 | PC2 | PC3 | PC1 | PC2 | PC3 | PC4 |
| As | **0.834** | -0.144 | 0.225 | -0.033 | -0.017 | **0.881** | -0.112 |
| Cd | 0.186 | -0.208 | **0.776** | **0.816** | -0.093 | 0.198 | 0.073 |
| Cr | **0.824** | 0.265 | -0.156 | -0.453 | 0.292 | 0.104 | **-0.609** |
| Cu | 0.362 | **0.781** | 0.203 | **0.610** | **0.490** | -0.202 | -0.232 |
| Ni | 0.265 | **0.641** | -0.311 | -0.104 | 0.124 | 0.047 | **0.870** |
| Pb | -0.093 | **0.773** | 0.072 | 0.410 | 0.183 | **0.635** | 0.304 |
| Fe | **0.811** | 0.247 | 0.208 | -0.168 | **0.673** | 0.241 | -0.091 |
| Zn | 0.032 | **0.413** | **0.782** | 0.116 | **0.756** | -0.084 | 0.131 |
| Eigenvalue | 2.28 | 1.98 | 1.48 | 1.46 | 1.41 | 1.34 | 1.32 |
| Variance(%) | 28.5 | 24.8 | 18.4 | 18.3 | 17.6 | 16.7 | 16.5 |
