# Supplementary figures and images for: Heavy metal contamination in the complete stretch of Yamuna river: A fuzzy logic approach for comprehensive health risk assessment
Source: PLoS One. 2022 Aug 8;17(8):e0272562. doi: 10.1371/journal.pone.0272562 (PMC9359575; doi:10.1371/journal.pone.0272562)

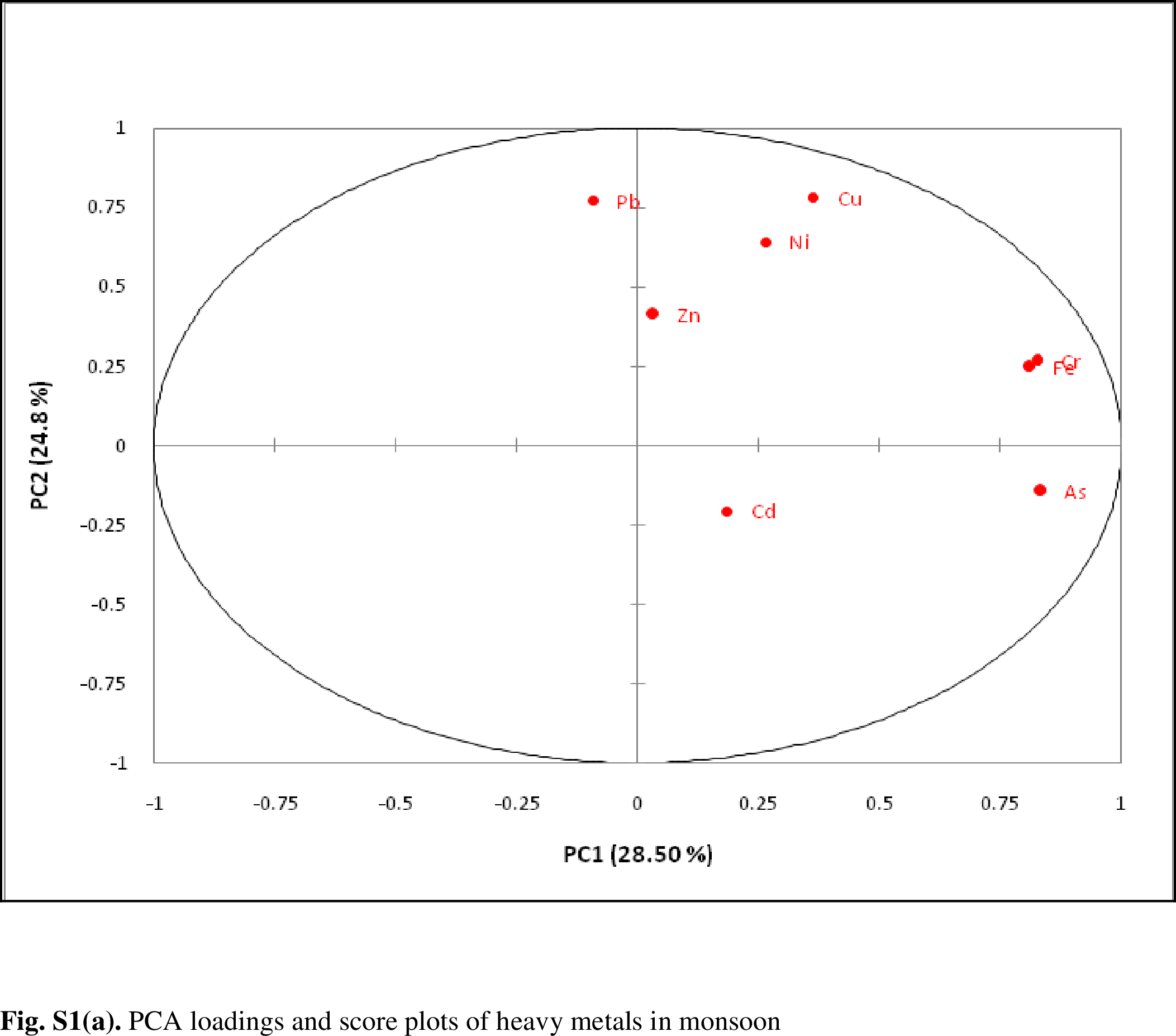

Supplement: S1 Fig — (TIF) [file pone.0272562.s007.tif]

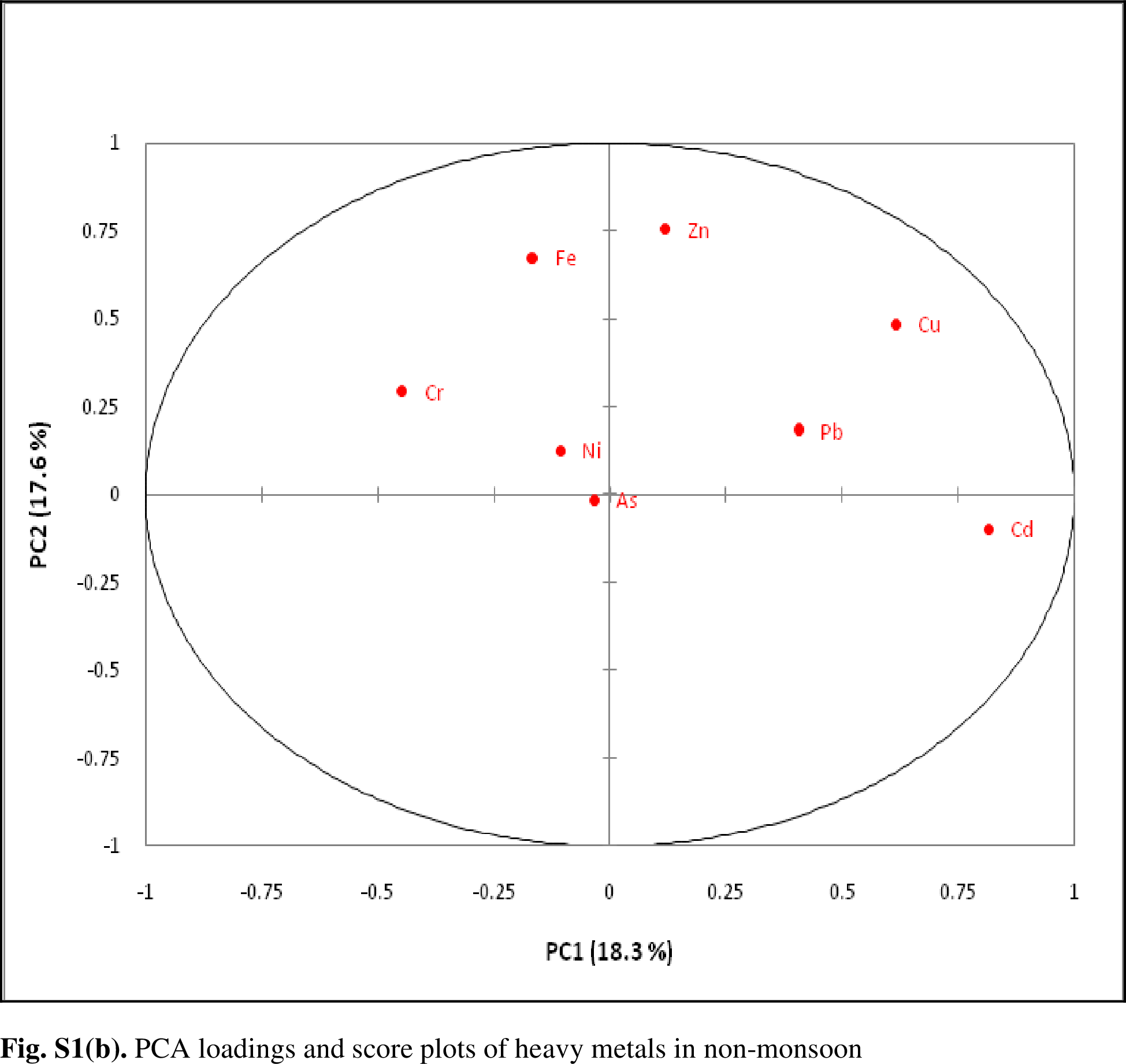

Supplement: S2 Fig — (TIF) [file pone.0272562.s008.tif]

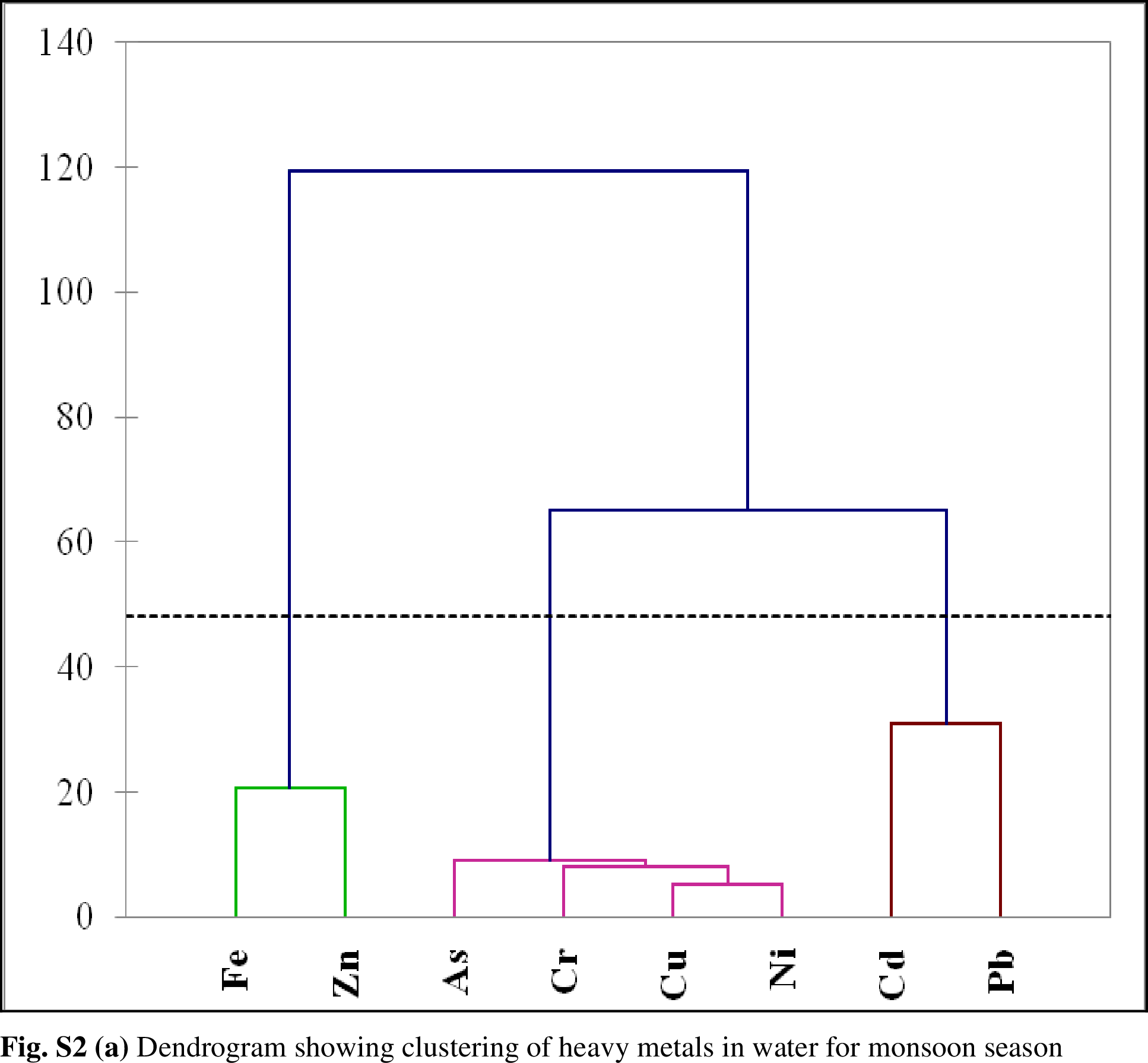

Supplement: S3 Fig — (TIF) [file pone.0272562.s009.tif]

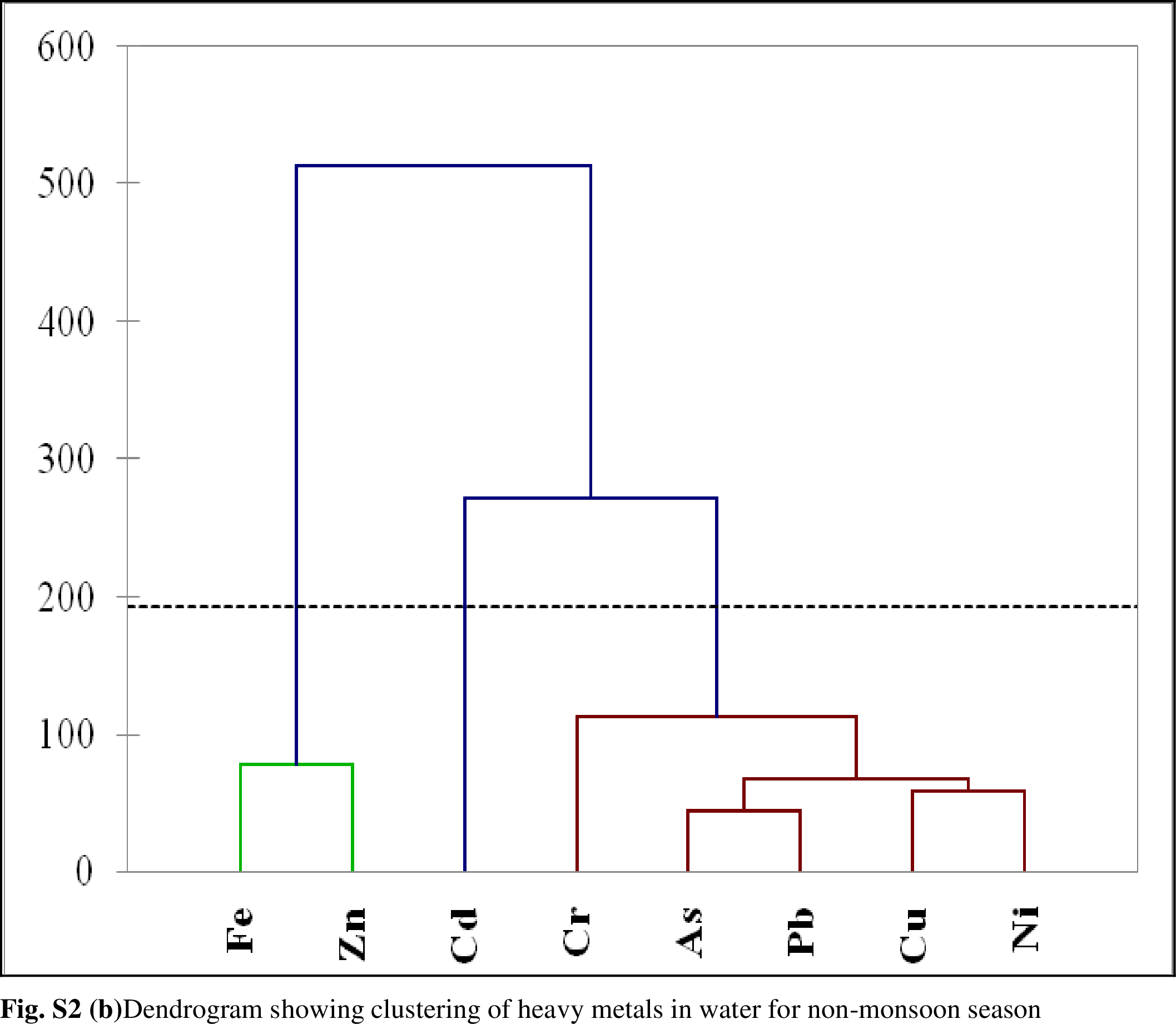

Supplement: S4 Fig — (TIF) [file pone.0272562.s010.tif]
